# Repeated sleep deprivation selectively reactivates hippocampal CA1 pyramidal neurons

**DOI:** 10.1186/s13041-026-01298-y

**Published:** 2026-04-01

**Authors:** Yutong Wang, Emily N. Walsh, Junko Kasuya, Colton E. Remedies, Jon Resch, Lisa C. Lyons, Ted Abel

**Affiliations:** 1 Department of Neuroscience and Pharmacology, Carver College of Medicine, University of Iowa, 51 Newton Road, 2-417B Bowen Science Building, Iowa City, IA 52242 USA; 2 Iowa Neuroscience Institute, Carver College of Medicine, University of Iowa, 169 Newton Road, 2312 Pappajohn Biomedical Discovery Building, Iowa City, IA 52242 USA; 3 Interdisciplinary Graduate Program in Neuroscience, 356 Medical Research Center, University of Iowa, Iowa City, IA 52242 USA; 4 Program in Neuroscience, Department of Biological Science, Florida State University, 319 Stadium Drive, Tallahassee, FL 32306 USA; 5 Present Address: Department of Neurology, Washington University School of Medicine, 660 South Euclid Avenue, St Louis, MO 63110 USA

**Keywords:** Sleep deprivation, Hippocampus, CA1, Pyramidal neurons, c-Fos, RiboTag, fosTRAP-seq

## Abstract

Sleep supports a variety of physiological processes, ranging from metabolic to immune system homeostasis, and plays a critical role in cognition and memory. A brief period of sleep loss impairs memory, particularly hippocampus-dependent memories, alters molecular signaling, and synaptic plasticity in the hippocampus. Studies have shown that sleep deprivation (SD) alters neuronal activation as indicated by broad changes in gene expression signatures and by the increased expression of c-Fos, an immediate early gene that functions as a molecular marker of neuronal activity. In the present study, we examined hippocampal subregion-specific c-Fos induction patterns via immunohistochemical staining. We found that CA1 pyramidal neurons exhibit the most robust c-Fos induction after SD. Using an activity-driven ribosomal tagging system (c-Fos-RiboTag) and a repeated SD model, we labeled sleep deprivation activated CA1 neurons and observed a population of excitatory neurons in area CA1 that are reactivated by repeated SD. Using the c-Fos-RiboTag system that enables the isolation of ribosomes with attached mRNA from labeled neurons, we performed fosTRAP-seq and identified activity-dependent gene expression changes in c-Fos+ CA1 neurons. Our results revealed that synapse organization, protein dephosphorylation, cellular response to endogenous stimulus (such as insulin) are upregulated, whereas mRNA processing and splicing are downregulated. In summary, our study provides a detailed characterization of hippocampal neuronal activation following SD and identifies a subset of CA1 pyramidal neurons that are selectively reactivated by repeated SD. This SD-sensitive neuronal population enables investigation of molecular changes in neurons specifically impacted by sleep loss and suggests a potential connection between acute and chronic sleep loss at the cellular and molecular levels.

**Graphical Abstract:**

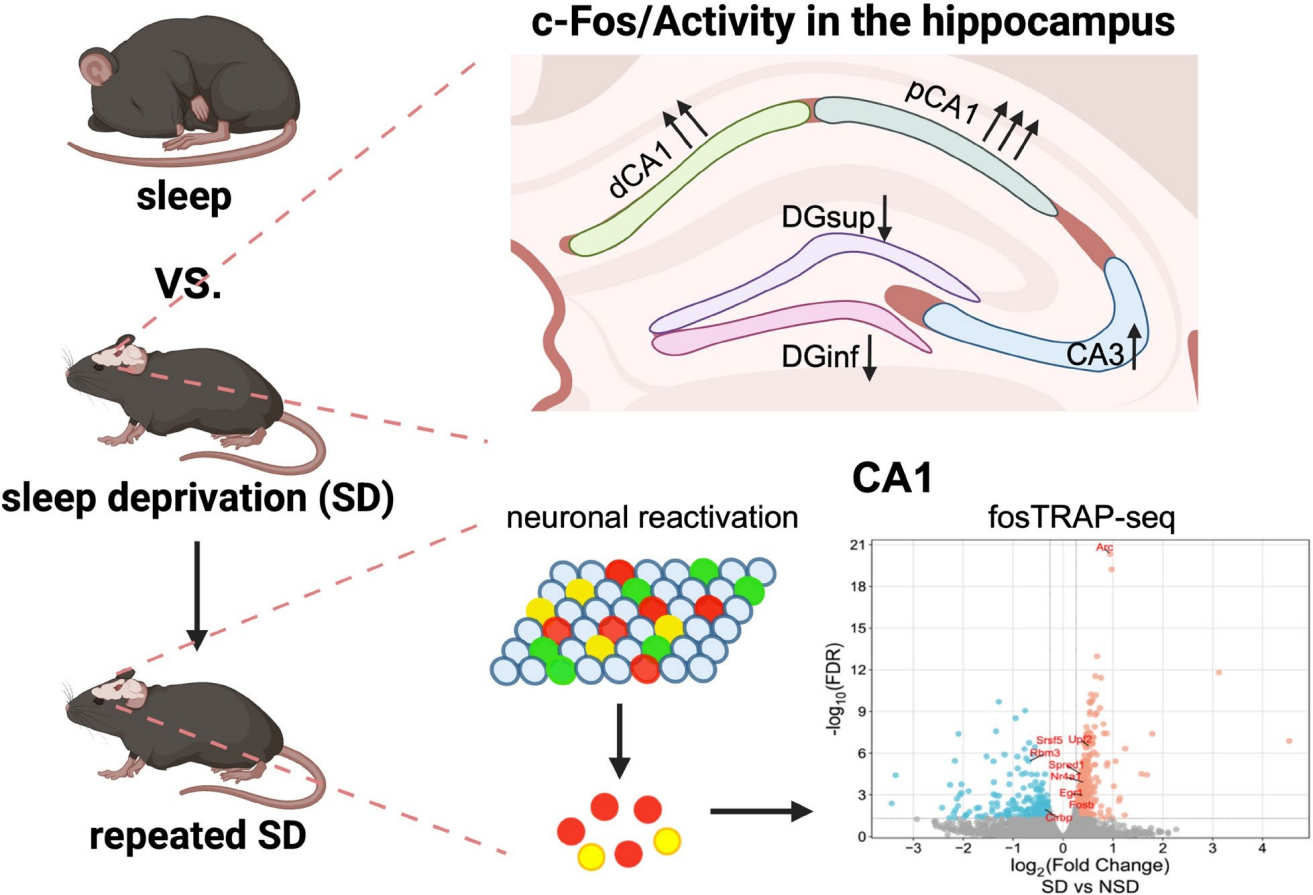

**Supplementary Information:**

The online version contains supplementary material available at 10.1186/s13041-026-01298-y.

## Introduction

Sleep facilitates numerous physiological processes including cognition, learning, and memory consolidation [1]. Over 30% of adults in the United States routinely experience insufficient sleep, leading to subsequent health problems [2, 3]. Sleep loss has pronounced impacts on cognitive function, with hippocampus-dependent memories being especially vulnerable [4]. The hippocampus plays a critical role in consolidating spatial, episodic, and contextual memories in rodents [5] and declarative memory in humans [6]. It is comprised of subfields with specialized roles in memory: the Dentate Gyrus (DG) enables pattern separation [7], Cornu Ammonis 3 (CA3) integrates separation and completion [8], and Cornu Ammonis 1 (CA1) recodes CA3 outputs while linking to the neocortex for memory retrieval [9]. Brief periods of sleep deprivation (SD) impact hippocampal function via impairments in the cAMP signaling and related long-lasting forms of synaptic plasticity [10–12], suppressed protein synthesis [13], and altered synaptic connectivity [14–16].

In humans, sleep loss can often span several nights or even weeks. Chronic insomnia is related to accelerated cognitive decline and is associated with increased risk of dementia in humans [17, 18]. Work using rodents to model chronic sleep restriction (CSR) has demonstrated impairment of hippocampal synaptic plasticity and severe spatial memory deficits that are resistant to recovery sleep [19, 20]. CSR also promotes tau hyperphosphorylation and amyloid-β accumulation in rodent models of Alzheimer’s Disease [21, 22]. However, how repeated bouts of sleep loss affect neuronal activity in the hippocampus and how acute SD-induced impairments progress to CSR-promoted neurodegeneration remains unknown.

A widely recognized marker of neuronal activity is the immediate early gene (IEG) c-Fos. The c-Fos protein is rapidly activated during synaptic plasticity associated with learning and memory [23], and its downstream effector genes, such as *Timp1* and *Mmp9*, regulate the neuronal structural and functional plasticity required for memory formation [23]. c-Fos expression has been used as a marker to locate and identify the neuronal populations activated by sleep and extended wakefulness [24–27]. While sleep is typically linked to low c-Fos expression and neuronal activity across most brain regions [24, 25], several hours of SD significantly increases c-Fos expression, particularly in the cortex, medial preoptic area, posterior hypothalamus, and the hippocampus [24–27]. This brain-wide activation resembles the increased c-Fos expression pattern during spontaneous wakefulness. Given the anatomical and functional complexity of the hippocampus, this study aims to precisely map c-Fos expression patterns within hippocampal subregions after single and repeated SD exposures, and identify activity-driven gene expression changes within SD-activated neurons.

Our results suggest that neuronal activation, as measured by c-Fos expression, varies across the hippocampus following both single and repeated SD. Specifically, the CA1 subregion showed the most pronounced increase in activation, while the DG exhibited a decrease in c-Fos expression after both single and repeated SD. Applying the Target Recombination in Active Populations (fosTRAP) method in conjunction with the ribosome tagging strategy (RiboTag) [28, 29], we devised an active neuron labeling system that was amenable to analysis of individual gene expression. Through this c-Fos-RiboTag approach, we confirmed that a substantial proportion of CA1 pyramidal neurons were reactivated by repeated bouts of SD. As prior genomic studies identified translation-level changes in brain regions exhibiting increased c-Fos expression after SD, including the hippocampus [30–33], we combined the c-Fos-RiboTag labeling system with Translating Ribosome Affinity Purification sequencing (TRAPseq) technique and identified activity-dependent translatome changes in c-Fos+ CA1 neurons repeatedly activated by sleep deprivation.

## Methods and materials

### Animals

All experimental animals were 3- to 5-month-old male C57BL/6J mice obtained from the Jackson Laboratories (Catalog# 000664, Bar Harbor, ME). Mice were group housed (5 per cage) prior to the start of the experiment in standard ventilated cages with access to food (NIH-31 irradiated modified mouse diet #7913) and water *ad libitum.* Animals were maintained in the animal care facility at the University of Iowa on a 12 h light/dark cycle, in a temperature- and humidity-controlled environment (21–22 °C and 60–70%, respectively). All experiments were conducted in accordance with the standards established by the US National Institutes of Health Guidelines for Animal Care and use was approved by the Institutional Animal Care and Use Committee (IACUC) at the University of Iowa.

### Sleep deprivation

Mice were single housed in cages with corncob bedding and a handful of soft bedding for nest building for 7 days prior to SD or non-sleep deprivation (NSD). Mice had *ad libitum* access to food and water in these housing conditions and during the sleep deprivation experiments. Mice were habituated to the experimenter and gentle handling stimuli, which was done by the experimenter holding each mouse in the palm for 2 min and cage tapping for 2 min for 4 consecutive days prior to the 1st sleep deprivation bout. Total SD was performed acutely, for 5 h beginning at Zeitgeber time (ZT) 0 using the gentle handling method, which involved lightly tapping and shaking the cage to keep the animal awake [12]. After 5 h, mice were returned to the original standard housing room where NSD mice were kept undisturbed throughout the experimental time window (ZT0-ZT5). The second SD or NSD bout was performed a week later using the same gentle handling method and timeline. After the second bout of SD/NSD, mice went through perfusion fixation for immunohistochemistry validation of c-Fos/RiboTag expression or cervical dislocation to collect tissue for RNA extraction from the hippocampal CA1.

For active neuron tagging experiments: injection habituation occurred for 2 consecutive days prior to the first sleep deprivation experiment in which mice received an i.p. injection of saline after the 2 min of handling and before being placed back in the cage for 2 min of light cage tapping. All mice received an i.p. injection of 15 mg/kg or 50 mg/kg 4-hydroxytamoxifen (4-OHT) at ZT2 on the day of first SD/NSD.

### Adeno-associated virus (AAV) constructs and stereotactic surgeries

Surgeries were performed under isoflurane anesthesia (5% induction, 2% maintenance) following administration of Meloxicam (5 mg/kg subcutaneous, s.c.). Animal health was monitored for 5 days following surgery along with administration of Meloxicam (5 mg/kg, s.c.) for the first 2 days post-surgery. To label the SD activated hippocampal neurons, 10–12 week old mice were injected intrahippocampally with a cocktail of AAV8-c-Fos-ERT2-Cre-ERT2-PEST-WPRE (titer- 1.57E + 13 GC/mL, generated and packaged by Stanford University Gene Vector, catalog# GVVC-AAV-139) and AAV9-EF1a.DIO.HA-mRpl22.IRES2.eYFP.WPRE.hGH (titer- 1.14E + 13 GC/mL, generated and packaged by the University of Pennsylvania Viral Vector Core). To label cells activated by SD across hippocampal subregions, the AAV cocktail was initially injected into the dorsal hippocampus bilaterally (1000nL at a rate of 200nL/min) using the following coordinates relative to bregma: anteroposterior (AP) -1.9 mm, mediolateral (ML) ± 1.5 mm, dorsoventral (DV) -1.5 mm from the surface of the brain. This whole hippocampus injection coordinate was used to evaluate labeling time window and 4-OHT dosage (Fig. S1). Following these evaluation experiments, the injection coordinates were optimized for CA1-restricted labeling. The AAV cocktail was injected into dorsal CA1 bilaterally (300nL at a rate of 50nL/min), the following coordinates were used upon validation: AP -1.8 mm, ML ± 1.45 mm, DV -1.65 from the surface of the skull. This CA1- localized AAV infusion coordinate was used for subsequent experiments examining CA1 neuronal reactivation (Fig. 5) and translating mRNA extraction for fosTRAP-seq (Fig. 6). Experiments were performed after 3 to 4 weeks to allow recovery from surgery and for spread of the viral particles in the targeted brain regions.

### Drug preparation

4-OHT solution was prepared fresh on the day of experiment as previously described [34]. 4-OHT (Catalog #H6278, Sigma-Aldrich, MO, USA) was dissolved in 100% ethanol at 37 °C for 15 min with constant rotation to prepare a 20 mg/mL stock solution. Stock solution was then diluted to 10 mg/mL in 100% corn oil followed by another 15 min incubation at 37 °C with constant rotation. The solution was vacuum centrifuged for 15 min to evaporate the ethanol, yielding an injectable solution of 10 mg/mL 4-OHT in corn oil, and was administered intraperitoneally (i.p.) at a dose of 15 mg/kg or 50 mg/kg body weight.

### Immunohistochemistry (IHC)

Perfusion fixed brains (4% v/v paraformaldehyde in 1X phosphate buffer saline (PBS), Catalog #15710, Electron Microscopy Sciences, PA, USA) were equilibrated in sucrose solution (30%w/v in 1X PBS) for 48 h. The brains were then sliced into 30 μm sections using a Leica cryostat (Leica CM3050S, IL, USA) at -20 °C and stored in cryoprotectant solution (30% w/v sucrose, 30% v/v ethylene glycol, and 0.01% Sodium Azide in 1X PBS). Sections containing the dorsal hippocampus were rinsed three times for 5 min each in 1X PBS, followed by a 1 h incubation in blocking solution (3% normal Donkey/Goat serum in 0.4% triton-X100/PBS buffer) and an overnight incubation at room temperature (RT) with primary antibodies: rabbit anti-c-Fos (1:3000, #226008, Synaptic Systems, Goettingen, Germany), guinea pig anti-c-Fos (1:3000, #226308, Synaptic Systems), rabbit anti-HAtag (1:2000, #3724, Cell Signaling, MA, USA), mouse anti-NeuN (1:1500, #ab104224, Abcam, MA, USA), rabbit anti-Sox9 (1:1500, #ab185966, Abcam), mouse anti-GAD67 (1:1000, #MAB5406, Sigma-Aldrich). Following overnight incubation with primary antibodies, slices were rinsed three times for 5 min in 1X PBS and incubated with secondary antibodies: Alexa Fluor 488 goat anti-rabbit (1:1000, #A11008, Invitrogen, CA, USA), Alexa Fluor 647 donkey anti-rabbit (1:1000, #A32795, Invitrogen), Alexa Fluor 555 goat anti-guinea pig (1:1000, #150186, Abcam), Alexa Fluor 647 goat anti-guinea pig (1:1000, #A21450, Invitrogen), and Alexa Fluor 488 donkey anti-mouse (1:1000, #A21202, Invitrogen) for 2 h at RT followed by three rinses in 1X PBS for 5 min each. Slices were mounted onto Superfrost Plus (Fisherbrand) slides with Prolonged Diamond Antifade mountant with DAPI (P36962, Thermo Fisher Scientific, MA, USA) to stain cell nuclei.

### Confocal microscopy and image analysis

Following IHC, images of the dorsal hippocampus were acquired using a Leica SPE Confocal microscope equipped with lasers at 405 nm, 488 nm, 561 nm, and 635 nm. All images (8-bit) were obtained with identical settings for laser power, detector gain, and pinhole diameter (1.0AU) using a 20X oil immersion objective at 1024 × 1024-pixel resolution and 1.5X optical zoom.

The Adult Mouse Allen Brain Reference Atlas was used as a reference for the hippocampus and hippocampal subregions. A total of 2–3 sections (2 hippocampi per section) were imaged per mouse and averaged get the number of c-Fos puncta per animal. Images were processed using the ImageJ software. Background fluorescent signals generated from AAV infusion surgery was subtracted from c-Fos and HAtag channels using the Image Calculator function in ImageJ. Images were converted to 16-bit for c-Fos+ cell quantification. The c-Fos+ cells within the cell body layers of each subregion were then plotted and counted automatically using the Analyze Particle function in ImageJ. The HAtag+ cells, DAPI+ nuclei, and double-labeled with HAtag/NeuN/Sox9 cells were counted manually. For c-Fos and GAD67 overlapping comparison, GAD67 images were first merged with the DAPI channel and overlapping cells within cell layers were identified as inhibitory interneurons. Then, interneurons overlapping with c-Fos labeling were quantified as activated inhibitory interneurons in each subregion. The percentage of activated excitatory neuron in each subregion was calculated by subtracting the inhibitory (GAD67+) neuronal fraction from the neuronal (NeuN+) fraction and normalizing to the fraction of NeuN+ neurons.

### Immunoprecipitation and mRNA extraction

For translatome sequencing, mice were sacrificed at ZT5 by cervical dislocation after the 2nd SD/NSD. Hippocampi were rapidly dissected into ice-cold 1X PBS followed by microdissection of CA1 on ice under the Leica S4E stereo microscope as previously described [35]. CA1 tissues were collected in sterile, DNase and RNase-free tubes chilled on dry ice and immediately stored at -80 °C. Ribosome-associated mRNA was immunoprecipitated as previously described [29]. Both CA1 tissues pooled from 5 mice in each group were homogenized in 1.2mL homogenization buffer (50mM Tris pH 7.4, 100mM KCL, 12mM MgCl_2_, 10% Nonidet P-40 (NP40), 1mM DTT, 200U/mL Promega RNasin, 1 mg/mL heparin, 100 µg/mL cycloheximide, 1X protease inhibitor (#P8340, Sigma, MA, USA). The homogenates were centrifuged at 10,000x*g* for 10 min at 4˚C. The supernatant was incubated with 5µL Anti-HA.11 Epitope Tag Antibody (#16B12, Biolegend, CA, USA) for 4 h at 4 ˚C with rotation, followed by overnight incubation with Pierce Protein A/G magnetic beads (#88803, Pierce, MA, USA) at 4 ˚C with rotation. Supernatant was removed from the beads using a magnetic rack and the beads were washed 3 times with a high salt buffer (50mM Tris pH 7.4, 300mM KCL, 12mM MgCl_2_, 10% NP40, 0.5mM DTT, 100 µg/mL cycloheximide). mRNA bound to the beads were eluted at 4˚C in 350µL of RLT buffer from the RNeasy Micro Kit (#74004, Qiagen, Venlo, Netherland) supplemented with β-mercaptoethanol at 10µL/mL. DNAse digestion of IP-eluted mRNA were performed following Qiagen’s RNeasy extraction kit instructions. RNA concentration and quality was assessed using the Qubit™ RNA High Sensitivity Assay Kit (#Q32852, Thermo Fisher Scientific, MA, USA).

### Library preparation

RNA quality was assessed using an Agilent Bioanalyzer. RNA library preparation from samples of SD and NSD mice were prepared at the Iowa Institute of Human Genetics (IIHG), Genomics Division, using Illumina Stranded Total RNA Prep Ligation with Ribo-Zero Plus v01 sample preparation kit (Illumina, CA, USA). Pooled library was sequenced on the Element Aviti24 sequencer with 150-bp paired-end chemistry at the IIHG core.

### fosTRAP-seq data analysis

Data analysis was performed using Partek Flow interface (Partek, Illumina, CA). Following QA/QC, raw reads were subjected to the base trimming to remove poorly scored 3’-bases and aligned using STAR aligner version 2.7.8a [36]. Aligned reads were quantified by Partek E/M model and annotated to mouse reference genome mm39 Ensemble release 110. The resulting mRNA counts were filtered to exclude mRNA with minimum count ≤ 5 (1%) and normalized using median ratio method. Differential gene expression analysis was performed with DESeq2 [37]. Differentially Expressed Genes (DEGs) were identified by fold change > 1.2 or <-1.2, false discovery rate (FDR) value < 0.05.

Enrichment of upregulated/downregulated DEG-associated pathways from the fosTRAP-seq data was performed with a combination of Gene Ontology (GO-Biological Process-EBI-UniProt-GOA-ACAP-ARAP) and Kyoto Encyclopedia of Genes and Genomes (KEGG) database. The GO-BP analyses were performed with the clusterProfiler package in R [38] and the Cytoscape (version 3.10.4 https://cytoscape.org/) [39] plug-in ClueGo (version 2.5.10) [40]. Only the common GO-BP pathways between the two methods with an adjusted p value < 0.05 and gene counts ≥ 3 were considered as significant. Volcano plot of all DEGs and Cnet plots representing enriched GO-BP pathways with their associated DEGs were generated using SRplot website [41] (Fig. 6). KEGG analyses were performed using the Cytoscape plug-in ClueGo, pathways were considered significantly enriched with an adjusted p value < 0.05 and gene counts ≥ 3. To connect terms in the network, ClueGO utilizes kappa statistics in which here was set as ≥ 0.4 and generated the map figure of enriched functions (additional file1: Fig. S4, 5). For comparison between our fosTRAP-seq and previous excitatory TRAPseq data published by Lyons et al., [31] (Fig. 6), we first ran the same DEG identification protocol as described above on excitatory TRAPseq raw data and compared common DEGs between the two datasets. The quadrant diagram illustrating common DEGs (Fig. 6) were generated using GraphPad Prism v10. GO-BP analysis was performed to identify enrichment biological processes associated with common DEGs using clusterProfiler package in R [38] and the pathways with their associated genes were presented using a Sankey diagram generated using SRplot [41] as well. Biological functions associated with DEGs specific for each database were performed using KEGG analysis (additional file1: Fig. S5) with Cytoscape as described.

### Statistical analysis

**Fig. 1:**
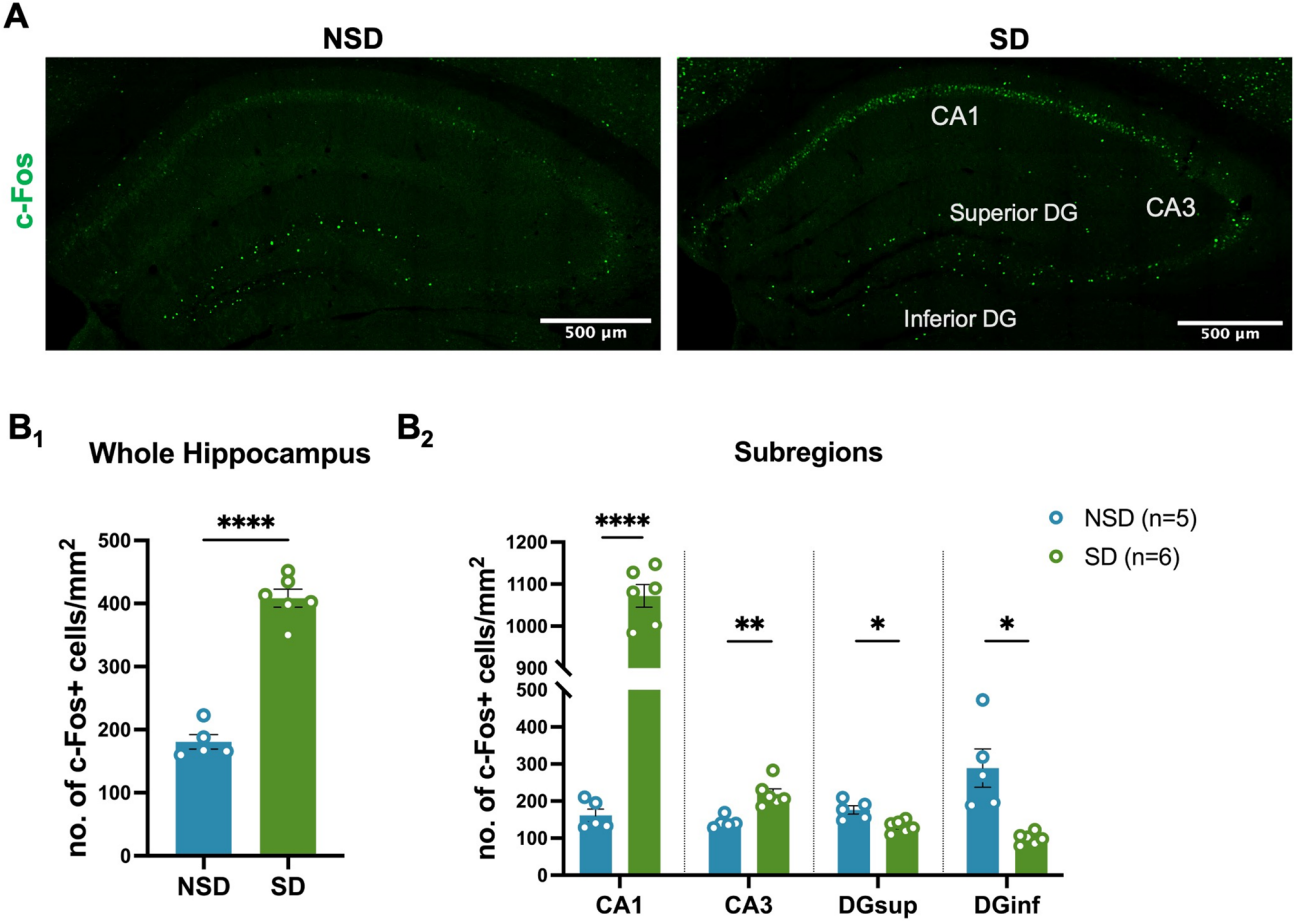
c-Fos expression is differently altered within hippocampal subregions after acute sleep deprivation. **A** Immunofluorescent images of c-Fos expression in NSD and SD hippocampus. **B** Comparison of area normalized c-Fos expression between NSD (*n* = 5) and SD (*n* = 6) groups from the entire hippocampus (1) and within different subregions (2): CA1, CA3, and superior and inferior blades of Dentate Gyrus (DGsup and DGinf). Data are shown as mean ± standard error of the mean (SEM). Unpaired two-tailed t test for B_1_: NSD = 180.64 ± 11.53, SD = 408.39 ± 14.27, t (9) = 12.05, *p* < 0.0001. Multiple unpaired t tests for B_2_: CA1: NSD = 161.50 ± 17.15, SD = 1072.02 ± 26.98, t (9) = 27.10, *p* < 0.00001; CA3: NSD = 142.53 ± 6.97, SD = 218.99 ± 14.11, t (9) = 4.54, *p* = 0.0014; DGsup: NSD = 176.18 ± 11.26, SD = 131.76 ± 6.58, t (9) = 3.56, *p* = 0.0061; DGinf: NSD = 289.11 ± 51.80, SD = 99.07 ± 6.38, t (9) = 4.02, *p* = 0.0030

All statistical analyses were performed using GraphPad Prism v10 with α = 0.05 for analyses. Imaging data comparing c-Fos and RiboTag expression and neuronal reactivation were analyzed using unpaired two-tailed t-tests to compare the NSD and SD groups (Fig. 1, 3C, 4, 5) and the distal and proximal CA1 sections (additional file1: Fig. S3). Two-way analysis of variance (ANOVA) comparisons were used to determine the interaction between c-Fos activation and number of sleep deprivation epochs (Fig. 3D, E). Data is presented as Mean ± SEM and *n* represents the number of animals in all experiments, IHC data is presented as an average of 2–3 sections per animal per condition. In figures, ns refers to non-significant, * refers to a *p* value < 0.05, ** refers to *p* < 0.01, *** refers to *p* < 0.001, and **** refers to *p* < 0.0001.

## Results

### Sleep deprivation causes non-uniform activation of hippocampal subregions as measured by c-Fos expression

Induction of IEGs, such as *Fos*,* Arc*,* and Egr1*, mark recent neuronal activation, and previous studies have investigated the impact of sleep and sleep loss on neuronal activity across rodent brains by measuring mRNA or protein levels of IEGs [24–27, 30, 32, 42]. To investigate the impact of acute SD on neuronal activation within hippocampal subregions, we sleep deprived mice for 5 h by the gentle handling method. This method was chosen to avoid confounds with methods that may cause novelty-induced hippocampal activation. Following 5 h SD, we mapped c-Fos protein expression, a well characterized marker of neuronal activity, within the hippocampus as an indicator of neuronal activation. Quantification of c-Fos protein abundance by immunohistochemistry in SD and NSD mice found increased c-Fos in SD samples suggesting an overall increase in hippocampal activity (Fig. 1A and B_1_) after 5 h of SD. As the hippocampal subregions have distinct functions, we quantified c-Fos expression within each hippocampal subregion finding that sleep deprivation for 5 h induced c-Fos expression in pyramidal cell layers in CA1 and CA3 subfields. However, we observed decreased c-Fos expression in the superior (DGsup) and inferior blades (DGinf) of the DG (Fig. 1B_2_). Furthermore, although CA1 and CA3 both showed an increase in c-Fos expression after 5 h of SD, the induction of c-Fos expression was more prominent in CA1 (8-fold induction compared to NSD) compared to CA3 (1.6-fold induction compared to NSD).

**Fig. 2:**
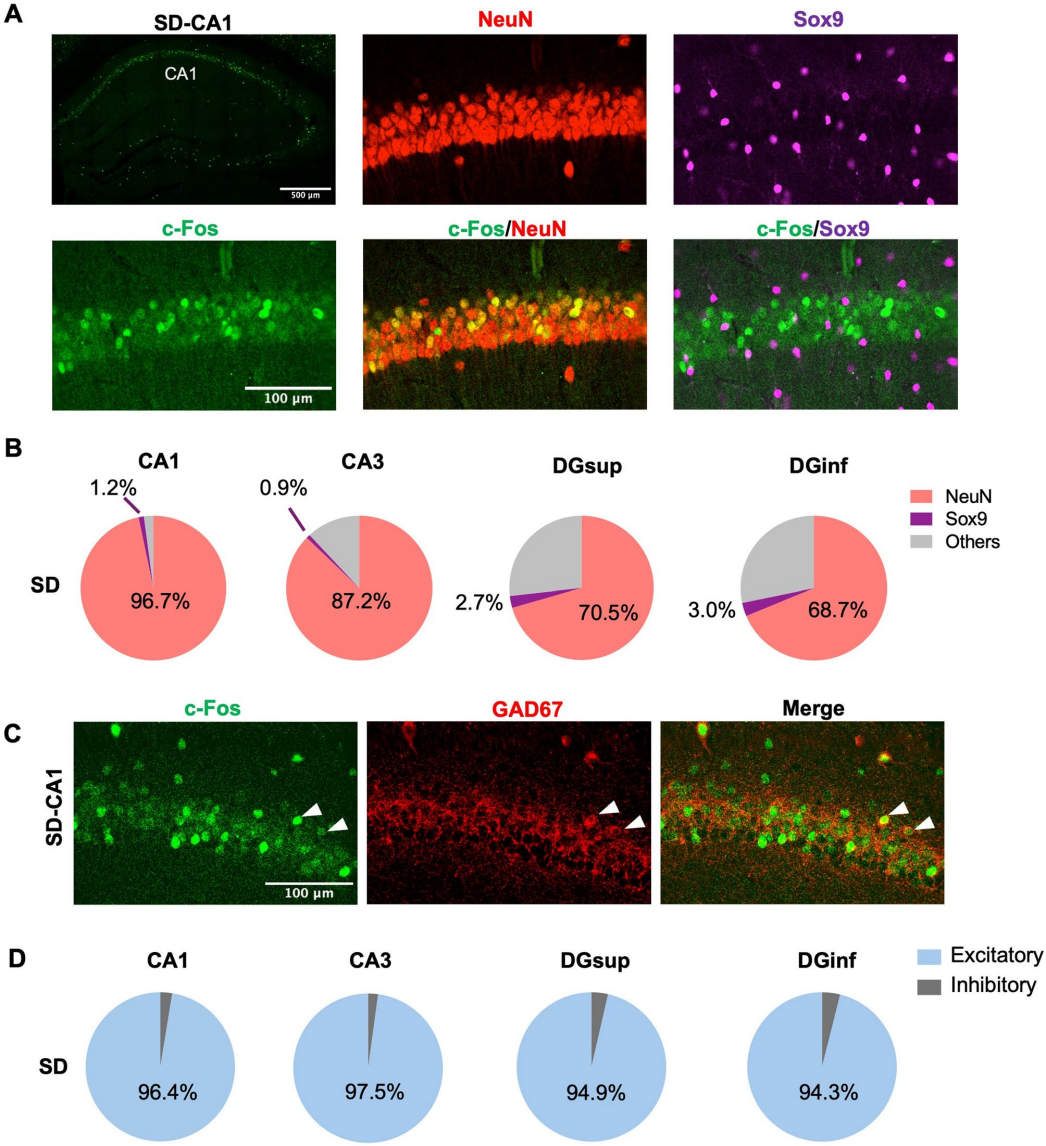
Excitatory neurons are selectively activated by SD. **A** Representative images of c-Fos double labeling with NeuN and Sox9 after SD. **B** Percentage of c-Fos+NeuN + and c-Fos+Sox9 + double labeled cells to the total number of c-Fos+ cells within each subregion in SD (*n* = 3) hippocampus. **C** Representative images of c-Fos double labeling with GAD67 (inhibitory neuronal marker) after SD. **D** Percentage of excitatory and inhibitory neurons to the neuronal population within cell layers of each subregion in SD (*n* = 3) hippocampus

We then asked whether a specific cell type within each subregion is selectively activated by sleep deprivation. To answer that question, we performed co-staining with c-Fos and cell-type specific markers (NeuN for neurons and Sox9 for astrocytes). Our results suggest that neurons (NeuN+) represent the majority of activated cells in all the subregions while a minor fraction of those c-Fos+ cells in each subregion are astrocytes (Sox9+) (Fig. 2A-B). In addition, depending on the subregion, a variable fraction of activated cells (c-Fos+) was classified as other cell types (e.g. microglia, oligodendrocytes) because they were negative for NeuN and Sox9. To further identify whether those c-Fos+ neurons are excitatory or inhibitory, we performed double labeling against c-Fos and GAD67. Our results indicate that sleep deprivation significantly activates excitatory pyramidal neurons (about 95% of all activated neurons) rather than inhibitory interneurons in all hippocampal subregions (Fig. 2C-D).

### Repeated SD has similar effects on c-Fos expression

**Fig. 3:**
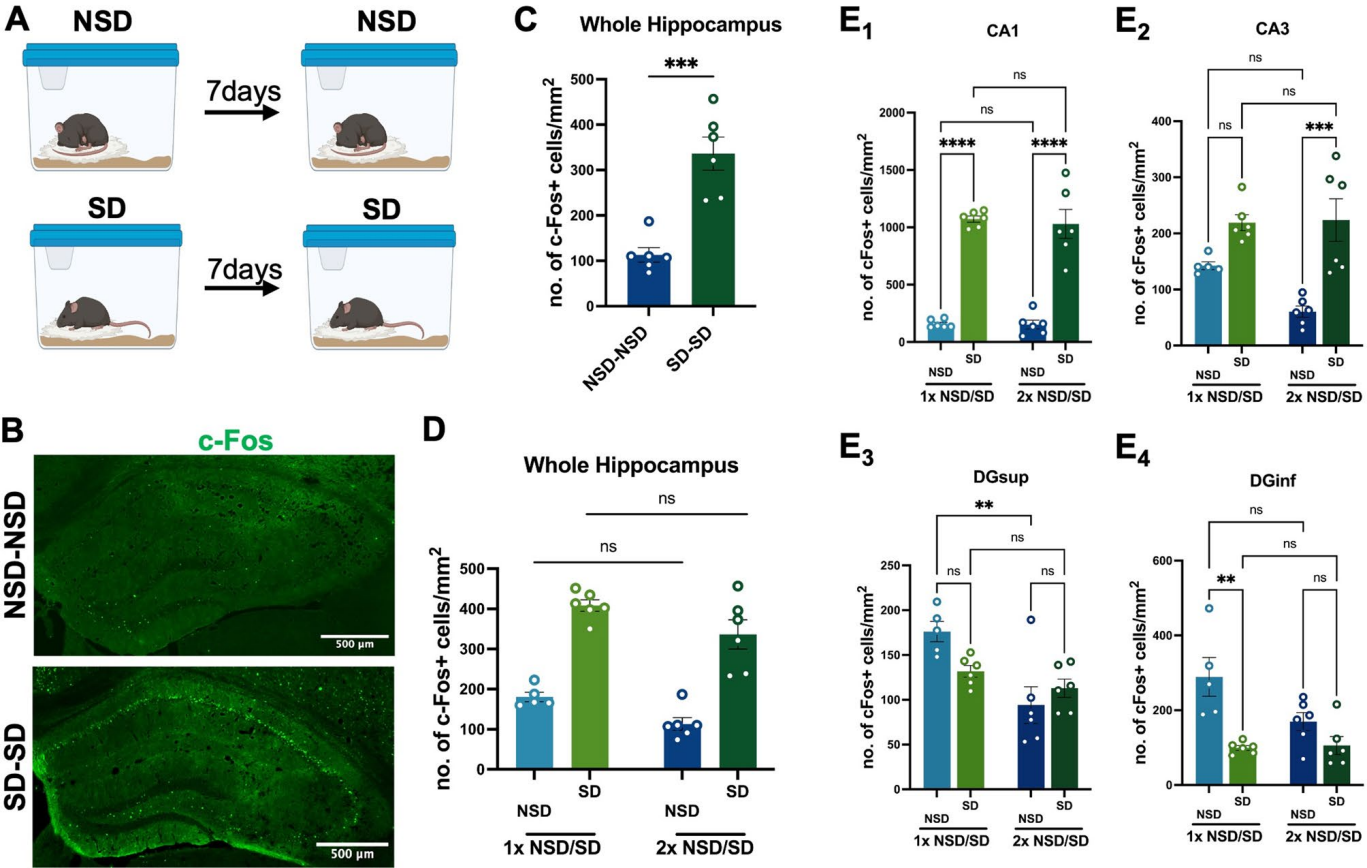
Repeated sleep deprivation induces a consistent pattern of c-Fos expression in the hippocampus similar to a single bout of SD. **A** Schematic of repeated sleep deprivation. **B** Immunofluorescent images of c-Fos expression after repeated NSD (NSD-NSD) and repeated SD (SD-SD) in the hippocampus. **C** Comparison of area normalized c-Fos expression between NSD-NSD (*n* = 6) and SD-SD (*n* = 6) whole hippocampus. Unpaired two-tailed t-test: NSD = 113.13 ± 15.84, SD = 336.38 ± 36.42, t (10) = 5.62, *p* = 0.0002. **D**,** E** Comparison of area normalized cFos expression between single (1x NSD/SD) and repeated sleep deprived (2x NSD/SD) hippocampus (**D**) and hippocampal subregions (**E**). Data are shown as mean ± SEM. **D**: Two-way ANOVA shows a significant effect of sleep deprivation (F (1, 19) = 98.43, *p* < 0.0001) and a significant effect of repeat manipulation (F (1, 19) = 9.42, *p* = 0.0063), but no significant effect of interaction (F (1, 19) = 0.01, *p* = 0.9224). **E**: Two-way ANOVA for each subregion: E1: effect of SD (F (1, 19) = 158.5, *p* < 0.0001), ns for effect of repeat manipulation and interaction; E2: effect of SD (F (1, 19) = 29.29, *p* < 0.0001), ns for effect of repeat manipulation and effect of interaction; E3: ns for effect of SD, effect of repeat manipulation (F (1, 19) = 14.23, *p* = 0.0013), effect of interaction (F (1, 19) = 5.57, *p* = 0.0291); E4: effect of SD (F (1, 19) = 19.45, *p* = 0.0003), ns for effect of repeat manipulation, effect of interaction (F (1, 19) = 4.81, *p* = 0.041). Bonferroni post hoc tests were performed for panel **D** & **E** to compare single vs. repeated exposure under SD & NSD conditions, as well as repeated NSD vs. SD c-Fos expression. Detailed 2-way ANOVA analyses results see additional file2: Table S1

In humans, SD is not usually a singular event, but frequently occurs on multiple nights or a weekly basis. However, how hippocampal neuronal activity is affected by repeated sleep loss remains poorly understood. To address this gap and to determine whether the prominent c-Fos induction in CA1 was due to stochastic neuronal activity or an inherent bias that makes a subset of neurons susceptible to sleep loss, we subjected animals to a repeated SD paradigm (Fig. 3A). In this paradigm animals were subject to SD twice with a 1 week-long recovery between the first and the second SD. This allowed us to determine how repeated SD alters c-Fos expression across hippocampal subregions. A second SD period revealed a similar pattern of c-Fos expression in the hippocampus as a single SD with induction in CA1 and CA3, and a decreasing trend in DG inferior blade compared to the NSD group (Fig. 3B, C). The repeated protocol itself does not have a significant effect on c-Fos abundance within subregions. We observed similar c-Fos expression levels between single and repeated NSD and single and repeated SD groups (1xNSD vs. 2xNSD, and 1xSD vs. 2xSD) within subregions, although the DG region showed decreased c-Fos after repeated NSD (Fig. 3D, E & additional file2: Table S1). These results indicate consistent levels of neuronal activation after each individual SD session. More importantly, the c-Fos induction patterns evident with a single SD were conserved after the second SD: high c-Fos induction in CA1 (7-fold) and a smaller induction in CA3 (3.5-fold).

Together, the results suggest that SD consistently induces region-specific changes in c-Fos expression and neuronal activation within the hippocampus, with the CA1 and CA3 regions showing increased c-Fos and the DG consistently showing decreased c-Fos expression.

### c-Fos driven RiboTag labels hippocampal neurons activated by sleep deprivation

Although there is a significant induction of c-Fos expression in CA1 pyramidal neurons after both single and repeated SD compared to NSD, the number of c-Fos+ neurons represent a small portion of the total number of CA1 pyramidal neurons. While the results from the repeated SD paradigm suggest that CA1 neurons are activated by SD, it is unknown whether the neurons within CA1 that show c-Fos induction are randomly recruited by individual SD sessions or whether neurons activated by an initial SD period are then more likely to be activated by additional periods of SD. To further characterize the neurons activated by repeated bouts of SD, we took advantage of the fosTRAP system, which can label neurons activated by defined stimuli [28].

**Fig. 4:**
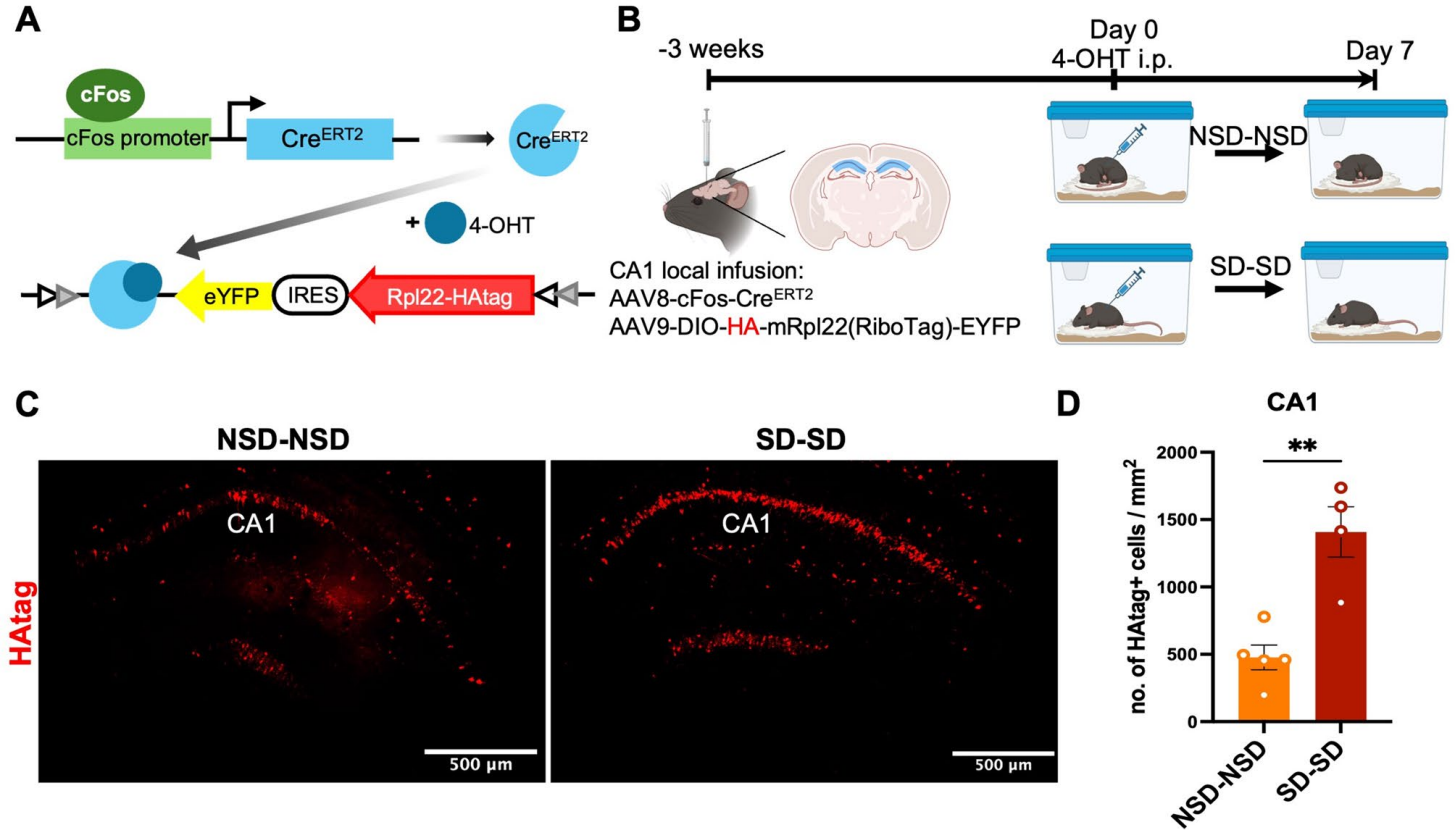
cFos-RiboTag strategy to label activated cells in area CA1. **A** Diagram of the activity (c-Fos)-driven ribosomal tagging (RiboTag) strategy. **B** Diagram of CA1 neuron labeling using c-Fos-RiboTag strategy with repeat sleep deprivation. **C** Representative images of neuron labeling shown as RiboTag (HAtag) expression in the NSD and SD hippocampus. **D** Neuronal labeling efficacy in hippocampal CA1: area normalized HAtag+ cells in NSD (*n* = 5) and SD (*n* = 4) groups. Unpaired two-tailed t-test performed, NSD = 476.91 ± 02.01, SD = 1408.06 ± 186.79, t (7) = 4.79, *p* = 0.0020. Data shown as mean ± SEM

We employed an AAV-based neuron labeling system in which animals were injected intrahippocampally with a cocktail of a c-Fos driven Tamoxifen-dependent Cre vector (AAV8-Fos-ERT2-Cre-ERT2) and a Cre-dependent RiboTag vector (AAV9-EF1a-DIO-HA-mRpl22-IRES-eYFP). Administration of 4-OHT facilitates expression of the tamoxifen-inducible c-Fos-driven Cre recombinase (Cre^ERT2^) upon neuronal activation, which subsequently drives expression of mRpl22-HA (RiboTag) [28, 29] (Fig. 4A) to label CA1 pyramidal neurons. Using this strategy, we first tested CA1 neuronal labeling efficacy, indicated by RiboTag (HAtag) expression, using different labeling durations, 4-OHT doses, and AAV infusion methods (additional file1: Fig. S1 and Fig. 4).

Unlike c-Fos protein itself, which is expressed rapidly following neuronal activation, expression of the c-Fos activation-promoted RiboTag depends on Cre^ERT2^ mediated recombination of loxP sites resulting in subsequent expression of mRpl22-HA (RiboTag) in activated neurons. We first assessed the amount of time needed to get robust expression of HA following 4-OHT delivery and SD or NSD. In a 5 h labeling test (additional file1: Fig. S1A-C), where 4-OHT was administered at ZT0 and tissue was collected after SD or NSD (ZT0-5), we observed only weak expression of the HAtag, and there were no differences between the SD and NSD groups, confirming that the RiboTag marker takes longer to develop. To provide a longer expression window, we waited one week based on previous results showing that repeated sleep deprivation leads to c-Fos activation in CA1 and CA3 that are comparable to a single SD (Fig. 3). Consequently, this approach requires the use of c-Fos-driven RiboTag labeling for neurons activated by an initial period of SD and subsequent c-Fos immunohistochemistry to identify neurons activated by a second period of sleep deprivation. We divided mice into repeated sleep deprivation (SD-SD) or repeated sleep (NSD-NSD) groups. All mice received an injection of 4-OHT at ZT2 of the 1st SD/NSD to drive RiboTag expression in neurons activated during the 5 h of SD/NSD [34]. We also tested two concentrations (15 mg/kg and 50 mg/kg) of tamoxifen and found that a higher dose of 4-OHT (50 mg/kg) was necessary to reflect the proportion of cells activated by SD (additional file1: Fig. S1D-F).

To further optimize RiboTag expression, we targeted the AAV cocktail infusion to hippocampal area CA1 bilaterally. Specific CA1 expression revealed a 3-fold induction of RiboTag expression after SD compared to the NSD group (Fig. 4). The expression level of RiboTag using this CA1 targeting strategy was higher than the number of cells showing c-Fos expression that we observed in both NSD and SD for the single or repeated SD groups (Figs. 1 and 3), particularly for the NSD groups. This could be due to the short half-life (~ 2 h) of c-Fos [43], and consequently the c-Fos protein that was induced in the early stages of the light cycle may not be reliably detectable at ZT5 using IHC in both the NSD and SD conditions, whereas the RiboTag will persist upon neuronal activation. Another factor that is likely to increase RiboTag expression in the NSD group is the unavoidable wakefulness and subsequent c-Fos expression induced by i.p. injection of 4-OHT. Together, these optimization strategies established a method for activity-driven RiboTagging that successfully labels CA1 pyramidal neurons that are activated by SD.

### CA1 pyramidal neurons are reactivated by repeated SD

**Fig. 5:**
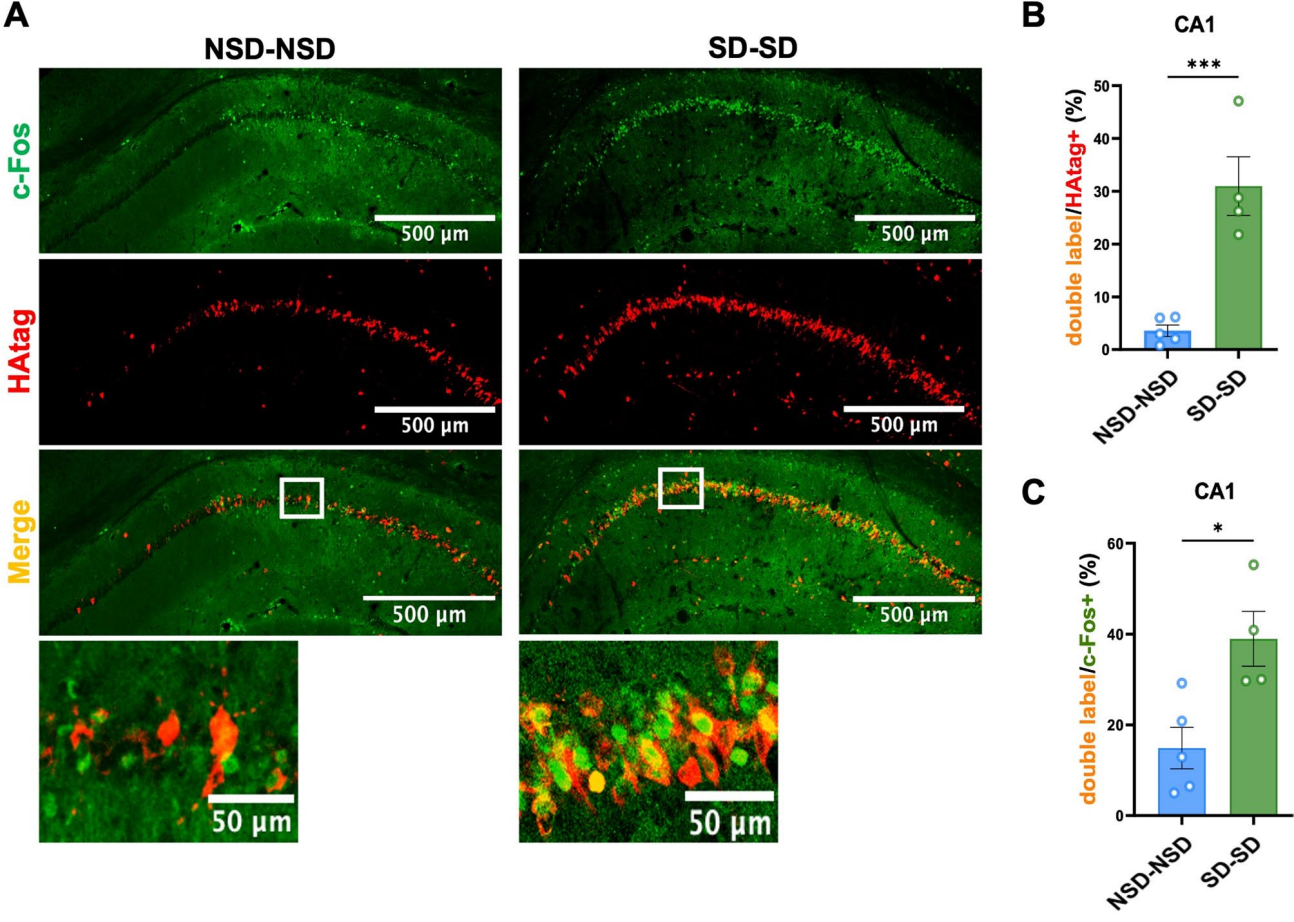
CA1 pyramidal neurons are reactivated by repeated SD. **A** Immunofluorescence images of c-Fos and HAtag double labeling in area CA1 in NSD-NSD and SD-SD groups. **B**,** C** Neuronal reactivation: percentage (%) of double labeled cells / HAtag+ cells (**B**) and double labeled cells / cFos+ cells (**C**) overlap in NSD-NSD (*n* = 5) and SD-SD (*n* = 4) CA1. Unpaired two-tailed t-test. Data shown as mean ± SEM. B: NSD-NSD = 8.82 ± 2.71, SD-SD = 30.97 ± 5.56, t (7) = 5.44, *p* = 0.0064. C: NSD-NSD = 36.02 ± 7.95, SD-SD = 38.96 ± 6.03, t (7) = 3.26, *p* = 0.7866

To answer the previous question of whether subsets of neurons reactivate during repeated SD in the CA1 or whether hippocampal CA1 pyramidal neurons are randomly activated by repeated sleep loss, we performed RiboTag combined with c-Fos immunolabeling. Immunofluorescence was used to identify the cells positive for RiboTag (the neurons that were activated during first SD or NSD) and c-Fos (the neurons that were activated during the second SD or NSD). Cells that are positive for both c-Fos and the HA tag are neurons that undergo reactivation during the second SD period (Fig. 5A). Our analysis showed that about 30% of CA1 neurons activated by the first SD (HAtag positive) were reactivated by the second SD (c-Fos positive; Fig. 5B). Furthermore, of the CA1 pyramidal neurons activated by the second SD (c-Fos+), 40% were previously activated by the first SD (Fig. 5C). To test whether the elevated reactivation observed in the SD-SD group could be explained solely by random overlap due to increased neuronal activation by SD, we calculated the expected overlap (E) under a random activation model in which activation during the second SD is independent of the first, such that the probability that a HA-tagged neuron expresses c-Fos equals the fraction of c-Fos+ neurons in the total cell population. Because the fraction of activated neurons is small, the majority of tagged neurons would be expected to remain c-Fos negative during the second SD if activation were random. Accordingly, expected overlap is calculated based on the number of HAtag + and c-Fos+ cells: $\:E=n\bullet\:\left(\frac{K}{N}\right)$, where K is the number of HA-tagged cells, n is the number of c-Fos+ cells, and N is the number of DAPI+ nuclei in the quantified CA1 region for each animal. We then calculated a reactivation index $\:RI=\frac{O}{E}$, where O is the observed number of double-labeled cells. The RI was 2.23 ± 0.24 (mean ± SEM, *p*=0.014 one-sample t-test) (addition file2: Table S2), indicating that the observed overlap during repeated SD is over 2-fold higher than the probability of random overlap. These results support non-random reactivation of SD-sensitive neurons across repeated SD exposures. To further examine whether the reactivation depended on SD history and re-exposure, we compared overlap across NSD-NSD, NSD-SD, SD-NSD, and SD-SD conditions (additional file1: Fig. S2). We first confirmed that the sleep state during the first episode did not affect neuronal activation during the second episode, i.e. the second SD induced a similar number of c-Fos+ cells regardless of whether mice experience SD or NSD during the first episode (Fig. S2B). Furthermore, the second SD did not elevate the number of neurons labeled by HAtag from the first SD episode (Fig. S2C). Overlap was highest in SD-SD animals and was significantly lower when SD occurred only once (NSD-SD) or when SD was not present during the second exposure (SD-NSD), indicating that the robust neuronal reactivation we observed require the second round of SD.

We also examined HAtag and c-Fos immunolabeling within the CA1 and found higher overall HAtag and c-Fos expression in proximal CA1, where pyramidal neurons are mainly driven by medial entorhinal cortex projections and involved in spatial information processing [44, 45]. Similar reactivation rates with subsequent SD were observed between the proximal and distal CA1 (additional file1: Fig. S3), which aligns with impaired spatial memory.

Overlap in c-Fos and HAtag was minimal in the NSD-NSD group: about 5% of RiboTagged cells during 1st NSD were activated by the 2nd NSD (Fig. 5B), and double-labeled cells make up approximately 15% of the limited population of c-Fos+ cells after the second NSD (Fig. 5C). This overlap is likely due to background RiboTag expression (additional file1: Fig. S1G).

Together, our results confirm that repeated SD results in a greater likelihood that CA1 pyramidal neurons originally activated by SD will be reactivated across repeated SD, suggesting that repeated SD stimulates a similar population of pyramidal neurons in the hippocampal CA1 rather than random neuronal activation.

### Activity-dependent translatome changes in SD-sensitive CA1 neurons

**Fig. 6:**
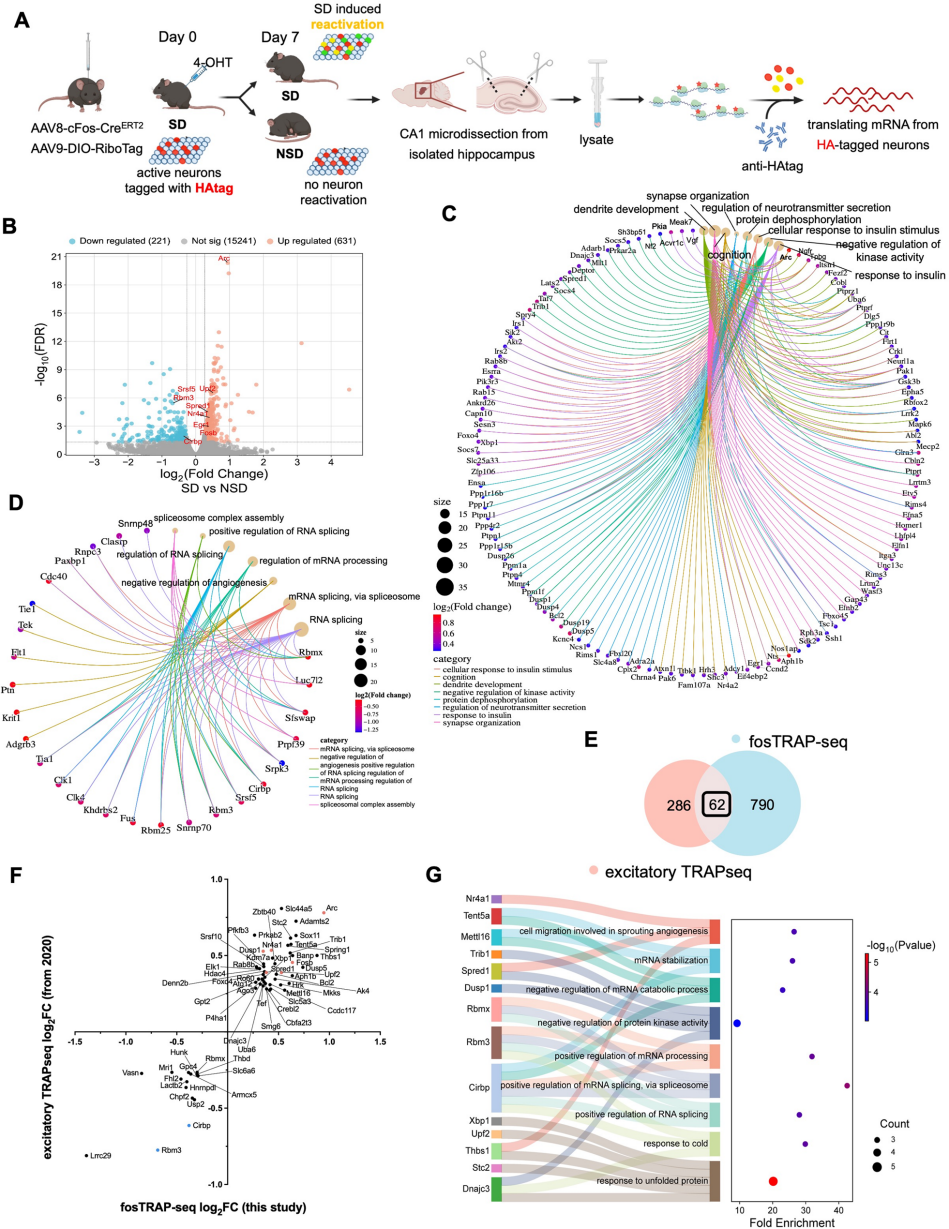
fosTRAP-seq reveals activity-dependent gene expression alteration in SD-sensitive hippocampal CA1 neurons after sleep deprivation. **A** Diagram of translating mRNA extraction from RiboTag labeled CA1 neurons. All mice were sleep deprived on day 0 then randomly divided into NSD and SD groups on day 7. Red: HAtagged neurons from 1st SD; Green: neurons activated during 2nd SD; Yellow: HAtagged neurons reactivated during 2nd SD. *n* = 10 mice/group, CA1 from 5 mice were pooled together for further RNA extraction. **B** Volcano plot of all DEGs (FDR < 0.05, |FC|>1.2) identified in response to sleep deprivation in hippocampal CA1 SD-sensitive neurons. **C**,** D** Cnet plot showing GO biological process (BP) analyzed pathway enrichment (adjusted *p* < 0.05, gene ≥ 3) associated with DEGs significantly upregulated (vull**C**) or downregulated (**D**) after sleep deprivation in SD-sensitive CA1 neurons. Full details of C, D see additional file2: Table S4. **E** Venn diagram representing the number of DEGs overlapped between our fosTRAP-seq results and our previously published (Lyons et al., 2020) excitatory TRAPseq after SD identified 62 common genes, 790 only in fosTRAP-seq and 286 only in excitatory TRAPseq. **F** Quadrant diagram showing comparison of common DEGs between fosTRAP-seq and excitatory TRAPseq. **G** Sankey diagram (left) shows association of overlapped DEGs to the identified GO biological processes (adjusted *p* < 0.05), along with pathway enrichment values as a Dot plot (right). Full details of **G** see additional file2: Table S7

In addition to tagging activated neurons, the RiboTag approach we used also enables the analysis of gene expression and mRNA translation in labeled cells, in our case the activated c-Fos+ neurons, through Translating Ribosome Affinity Purification with RNA sequencing (fosTRAP-seq) [29, 31]. We tagged SD-activated CA1 neurons by subjecting all mice to sleep deprivation on day 0, followed by division into NSD and SD groups on day 7 to induce changes in activity and gene expression (Fig. 6A). Immediately following the second SD or NSD session, actively translating mRNAs from CA1 RiboTag-labeled neurons were isolated using immunoprecipitation with antibodies for the HAtag. This paradigm enables gene expression comparisons within previously tagged SD-activated CA1 neurons under SD and NSD conditions. Following TRAP RNA extraction (5 animals pooled per sample), we used an unbiased RNA sequencing approach, aligned raw reads using STAR aligner [36], and identified differentially expressed genes (DEGs) by the criterion of: FDR < 0.05, and fold change > 1.2 or <-1.2 to avoid false positives. From our fosTRAP-seq data, we identified 631 DEGs being upregulated while 221 DEGs being downregulated in RiboTag labeled CA1 neurons (Fig. 6B, additional file2: Table S3). As expected, the IEGs (such as *Fosb*,* Arc*,* Egr1*,* and Homer1*) that are bound for translation are upregulated by sleep deprivation indicating reactivation of certain SD-sensitive CA1 neurons during the repeated sleep loss. We also observed decreased ribosome associated transcripts of *Cirbp*,* Rbm3*, *Srsf5* which have been reported by previous bulk transcription analysis [33, 46].

We then separately analyzed the upregulated and downregulated transcripts for the enriched biological processes and functions that were affected by SD against the GO: biological process (GO-BP) and KEGG databases. The analyses on the upregulated DEGs revealed strong enrichment in biological processes of protein dephosphorylation (e.g. *Dusp1/4/5/19/26*,* Ppm1a/1f*,* Ptpn1/4/11*), negative regulation of kinase activity (e.g. *Adarb1*,* Dnajc3*,* Pkia*,* Dusp1/19*), response to insulin (e.g. *Akt2*,* Foxo4*,* Gsk3b*,* Irs1/2*,* Capn10*,* Egr1*,* Tsc1*,* Vgf*,* Ptpn1/11*), dendrite development (e.g. *Arc*,* Cobl*,* Mack6*,* Mecp2*,* Ngfr*,* Ppp1r9b*), regulation of neurotransmitter secretion (e.g. *Adra2*,* Cplx2*,* Kcnc4*,* Lrrk2*,* Rim3/4*), cognition (e.g. *Arc*,* Egr1*,* Ccnd2*,* Nr4a2*,* Uba6*) having higher enrichment scores among all altered pathways (Fig. 6C, additional file2: Table S4-1), and the biological functions of longevity regulation and insulin signaling pathways (additional file1, 2: Fig. S4A, Table S5-1). The downregulated DEGs are mainly associated with regulation of mRNA processing and splicing processes (e.g. *Cirbp*,* Rbm3/x*,* Srsf5*,* Luc7l2*,* Cdc40*,* and Sfswap*), as well as negative regulation of angiogenesis (*Adgrb3*,* Flt1*,* Krit1*,* Ptn*,* Tek*,* and Tie1*) (Fig. 6D, additional file2: Table S4-2), and the biological functions of ECM-receptor interaction and spliceosome (additional file1,2: Fig. S4B, Table S5-2).

We also compared these data to our previous work performing TRAPseq [31] on bulk hippocampal excitatory neurons from the hippocampus after 5 h of sleep deprivation. There are 62 DEGs overlapped between our fosTRAP-seq and the excitatory TRAPseq and all DEGs share the same directional changes (Fig. 6E-F, additional file2: Table S6). Among the common DEGs, IEGs (including *Fosb*,* Arc*,* Homer1*,* Nr4a1*), *Upf* 2 (regulator of nonsense mediated mRNA decay), and *Spred1* (negative regulation of kinase activity/protein phosphorylation) are upregulated in both datasets. The *Cirbp* and *Rbm3* genes, involved in RNA splicing and mRNA processing regulation, are downregulated from the two datasets. GO-BP analysis indicates that protein phosphorylation, mRNA splicing and processing processes are shared in both datasets (Fig. 6G, additional file2: Table S7). Because neurons were tagged during the first SD and isolated after the second SD, the presence of these previously reported SD-altered genes suggests that the tagged neurons were reactivated and exhibited SD-associated gene expression changes during the second SD episode. This observation aligns with our cellular reactivation data and supports the interpretation that SD-activated neurons are repeatedly engaged by sleep loss. We also compared distinct DEGs between the two datasets and KEGG analysis revealed that biological functions of insulin-signaling pathway, longevity regulation pathway, and focal adhesion enriched specifically in our fosTRAP-seq, while melanogenesis and apoptosis are altered only in excitatory TRAPseq (additional file1,2: Fig. S5, Table S8). DEGs not reported in previous study may reflect molecular responses associated with repeated SD. The shared and unique DEGs between the two datasets further validate the reliability and robustness of our fosTRAP-seq method.

Taken together, our fosTRAP-seq enables subregion-specific analysis of activity-dependent translatome alterations in SD-vulnerable neurons. These alterations involve pathways related to post-transcriptomic and translational regulation, synapse connection, neuron development, and insulin signaling.

## Discussion

The hippocampus is a structurally distinct and functionally diverse brain region, comprised of interconnected subregions that contribute uniquely to memory encoding processes [7–9]. To better understand how sleep deprivation affects hippocampal function, we investigated c-Fos expression patterns across different hippocampal subregions. In this study, we sleep-deprived animals from ZT0-ZT5 by gentle handling and examined hippocampal subregional c-Fos expression after single and repeated SD. Results indicated that c-Fos expression is altered differently among hippocampal subregions: CA1, particularly proximal CA1, shows the most robust c-Fos induction; CA3 shows a moderate activity increase; and the DG shows reduced c-Fos expression. (Figures 1, 2 and 3). Using the c-Fos driven RiboTag labeling system, we observed 30% to 40% overlap ratio between neurons activated during the first and second SD and confirmed that CA1 pyramidal neurons are reactivated by repeated sleep loss (Figs. 4 and 5). We then performed fosTRAP-seq and revealed translatome changes in RiboTag labeled CA1 neurons that are activated by sleep deprivation (Fig. 6).

Sleep deprivation can be induced by different techniques, including gentle handling, novel object exposure, cage change, and automated moving treadmill or rotating wheel paradigms, each serving different experimental purposes and induces stress to different extent [47, 48]. Methods involving object and environmental novelty may engage hippocampal neuronal ensembles and influence activity-dependent markers such c-Fos. In contrast, the gentle handling protocol used here maintains wakefulness without introducing salient contextual stimuli or any direct interaction with the animal. Previous studies show that, following habituation, gentle handling does not significantly alter hippocampal plasticity, sleep/wake architecture, or basal stress hormone [49]. Although corticosterone increases have been reported after gentle handling induced SD, blocking corticosterone synthesis does not rescue SD-induced memory deficits, suggesting stress is not the primary driver of the memory impairments [50]. Consistent with this research, recent work demonstrates that gentle handling elicits less hippocampal c-Fos activation than novelty based deprivation methods [51]. Thus, we employed the gentle handling protocol to minimize potential confounding effects associated with stress, direct animal interactions and environmental novelty.

In the present study, we observed distinct patterns of neuronal activation among hippocampal subregions caused by sleep deprivation. Hippocampal neuronal activation is regulated by coordinated interactions among glutamatergic, GABAergic, serotonergic, and noradrenergic systems, and regional differences in receptor expression may account for distinct c-Fos activation patterns. During sleep deprivation, there are a multitude of changes in neurotransmitter systems and signaling that differ between areas of the cornu ammonis and dentate gyrus [52, 53]. Of note, locus coeruleus-derived norepinephrine release is elevated during SD and is known to induce c-Fos expression and enhance CA1 pyramidal neuron excitability via β-adrenergic signaling [54–56]. Consistent with this, our fosTRAP-seq data show that CA1 neurons are enriched for *Adrb1* relative to *Adrb2* and *Adrb3*, suggesting elevated β_1_-adrenergic receptor expression enhances sensitivity to norepinephrine and promotes c-Fos activation via cAMP-CREB signaling. This differential pattern of neuronal activation is consistent with previous spatial transcriptomic studies, which identified unique gene expression changes and enriched molecular functions specific to hippocampal subregions after SD [52, 57] and adds to a growing literature emphasizing the heterogeneity of SD effects in various hippocampal subregions. However, our findings differ from a recent study by Sarma and colleagues (2025), which reported that 3 h, but not 6 h, of gentle handling sleep deprivation alters c-Fos expression across hippocampal subregions [51]. In contrast, our 5 h sleep deprivation paradigm produced a more robust increase in c-Fos, particularly in CA1, which may reflect the higher sensitivity of immunofluorescence compared with DAB-based immunohistochemistry. Notably, our c-Fos expression pattern is consistent with prior genomic, translational, and behavioral studies showing upregulation of c-Fos and other IEGs after 5 h of sleep deprivation [31, 33, 46]. This same 5 h gentle-handling protocol reliably induces hippocampus-dependent memory deficits and synaptic plasticity alterations [12, 14] without elevating corticosterone or confounding behavioral stress effects [49, 50]. Together, these findings suggest that sleep-loss duration is a critical determinant of hippocampal c-Fos responses, with 5 h representing a key time point at which molecular, electrophysiological, and behavioral consequences of sleep deprivation converge.

Utilizing a viral c-Fos-RiboTag active neuron tagging approach [28, 29], we successfully captured the robust induction of CA1 neuronal activation and identified a population of CA1 pyramidal neurons that is repeatedly activated by sleep deprivation. Our approach labeled a substantial number of activated neurons compared to a previous study that reported minimal labeling of CA1 neurons following sleep deprivation using a fosTRAP transgenic mouse model [58]. Our results demonstrate over 30% overlap between c-Fos + and RiboTag-labeled neurons across repeated sleep deprivation events (Fig. 5). This represents a significant overlap of neuronal activation after repeated SD comparable to the hippocampal engram reactivation rate observed using IEG-dependent tagging methods (e.g. TRAP2 and Tet-tag) during memory consolidation and retrieval, which typically ranges from 5 to 20% [52, 59–61]. The reactivation of CA1 pyramidal neurons suggests the existence of a stable SD-responsive neuronal ensemble that can be reactivated by repeated bouts of SD. Unlike classical memory engrams that encode external experiences through activity-dependent synaptic strengthening, this population appears to represent a state-dependent neuronal population that is more likely to be reactivated by subsequent sleep loss. The reactivation of neurons across SD episodes raises the possibility that activation of those ensembles or subpopulations of neurons may mediate the molecular and functional consequences SD on hippocampal circuits.

c-Fos expression is widely used as indicator of engram activation and reactivation in hippocampus-dependent memory formation and consolidation. In CA1, higher neuronal place field preference, place field activity, stability of spatial maps, and more precise spatial information decoding is found in the neurons that were c-Fos positive during spatial learning tasks [62]. The strong activation of CA1 neurons during SD with c-Fos expression, despite the absence of learning, raises the question of the functional significance of CA1 neuronal activation under sleep loss. Several hours of sleep deprivation following learning similarly induces CA1 c-Fos expression and impairs memory consolidation [51, 52], implying that sleep loss may either recruit neuronal populations distinct from memory engrams or aberrantly activates learning-related ensembles while disrupting gene expression, protein synthesis, or sharp-wave ripple-mediated replay [63–65]. Future experiments comparing whether learning and sleep deprivation activates a similar population of CA1 pyramidal neurons could be helpful in answering this question. Together, these observations highlight a sleep deprivation-responsive CA1 ensemble subpopulation and provide a framework for dissecting how aberrant neuronal activation and reactivation contribute to hippocampal dysfunction and memory impairment under chronic sleep loss.

Taking advantage of the cFos-RiboTag labeling strategy with a repeated SD paradigm, we were able to isolate activity-dependent translatome alterations specifically in CA1 neurons activated by sleep loss (c-Fos+). Importantly, all animals experienced the initial SD episode before being assigned to either the second SD or NSD condition. Therefore, the molecular differences observed in our paradigm reflect responses to the second SD episode rather than residual effects from the initial SD exposure. Our fosTRAP-seq analysis identified DEGs that have been reported in previous gene expression studies after acute sleep deprivation [31, 33, 46, 57] supporting the robustness of our repeated SD and neuronal labeling strategy. We reported upregulated translation of transcripts involved in synaptic vesicle exocytosis (e.g. *Cplx2*,* Rims4*,* Slc4a8*), regulation of synaptic plasticity (e.g., *Arc*,* Egr1*,* Adcy1/8*), and dendrite development (e.g. *Arc*,* Cobl*,* Mack6*,* Mecp2*) all of which are essential for memory consolidation. In contrast, transcripts associated with mRNA splicing and processing (e.g., *Rbm3/25*,* Rbmx*,* Cirbp*,* Srsf5*,* Luc7l2*,* Tia1*,* Snrnp70*, *Fus*) were markedly downregulated by sleep deprivation. Activity-dependent protein synthesis is essential for synaptic remodeling and long-term plasticity required for memory encoding and engram stabilization [66, 67]. Disruption of those processes suggests that activation of those SD-responsive ensembles may not support adaptive memory encoding of sleep loss, but instead create a maladaptive molecular environment that interferes with hippocampal plasticity. Consistent with this idea, SD suppresses mTOR-mediated translation initiation and impairs LTP maintenance, which depends on protein synthesis, without affecting induction [10, 13]. In line with these findings, our fosTRAP-seq date reveal disrupted RNA processing and splicing in activated CA1 neurons, changes that could compromise the protein synthesis required to sustain synaptic plasticity and effective memory consolidation. Notably, brief recovery sleep restores IEG expression, protein synthesis, and synaptic plasticity [13, 14, 33], indicating that these alterations are reversible rather than simply a consequence of prolonged neuronal overactivation. Together, based on neuronal reactivation and our fosTRAP-seq data, we hypothesize that SD-activated CA1 neurons represent candidate SD-responsive ensembles that may contribute to the negative consequences of sleep loss through disrupting protein synthesis. However, direct causal testing will be required to determine their precise functional role.

Beyond previously reported DEGs identified from excitatory neurons from the entire hippocampus [31], we further detected (Fig. 6) increased translation of genes involved in insulin signaling and insulin resistance pathways, including *Akt2*,* Gsk3β*,* Irs1/2*,* Pik3r3*,* Ppp1r3c*,* Prkab2*,* Ptpn1/11*,* Ptprf*,* and Rps6ka2*. Brain insulin signaling regulates multiple cellular processes including glucose metabolism, cell survival, and synaptic plasticity through PI3K-Akt signaling and downstream phosphorylation of targets such as GSK-3β and mTOR [68]. Disruption of hippocampal insulin signaling such as developing insulin resistance impairs neuroplasticity and spatial memory in rats [69] and overactivation of GSK-3β has been linked to inhibition of hippocampal long-term potentiation [70]. Consistent with this framework, our fosTRAP-seq data indicate that insulin response pathways, particularly those relate to insulin resistance, are substantially altered in SD-sensitive CA1 neurons. These disruptions may serve as a central mechanism linking SD to interrupted synaptic plasticity, impaired signal transmission and reduced memory performance. Importantly, GSK-3β also participates in different aspects and pathways relevant to the onset and development of Alzheimer’s Disease neuropathology via directly promoting tau hyper-phosphorylation, amyloid production and accumulation, and inflammatory molecules [71]. Additionally, we observed increased translation of *Ttbk1/2* (tau tubulin kinase 1 and 2), kinases that phosphorylate tau at multiple sites and contribute to tauopathy in neurodegenerative disorders [72]. Given that chronic sleep restriction causes hippocampus-dependent memory deficits [73] and promotes tau hyperphosphorylation in both human and rodents [18, 21], our findings suggest a mechanistic continuum whereby repeated acute SD initiates insulin signaling dysfunction and upregulation of tau-related kinases (GSK-3β, TTBK1/2), which, when sustained, may accelerate tau pathology and neurodegeneration in CA1. Collectively, these results highlight insulin signaling dysfunction as a key molecular feature of SD-induced hippocampal impairment and propose a trajectory linking acute sleep loss to chronic sleep restriction-associated tauopathy and increased vulnerability to neurodegenerative disease.

There are several limitations that should be noted with the present study. Despite the minimal novelty induced by sleep deprivation using gentle handling compared with other paradigms, all sleep deprivation paradigms involve sustained wakefulness and ongoing sensory input, and no current method can fully dissociate the effects of sleep loss from accompanying arousal or environmental influences. Therefore, we cannot completely exclude contributions from method-related factors to the observed molecular changes. Additionally, although the present study identifies and molecularly characterizes a CA1 neuronal population that is selectively and repeatedly activated by SD, we did not directly test the behavioral or physiological consequences of manipulating these ensembles. Therefore, whether these SD-activated neurons are necessary or sufficient for mediating SD-induced cognitive and hippocampal impairments remains unresolved. Future studies combining activity-dependent neuron tagging with optogenetic or chemogenetic manipulation, together with electrophysiological and behavioral assays, will be required to establish the causal contribution of this SD-responsive ensemble to hippocampal dysfunction.

For future studies, a direct comparison of gene expression profiles between CA1 pyramidal neurons that are sleep loss sensitive (c-Fos+) and insensitive (c-Fos-) during SD will be important for delineating molecular features that confer selective vulnerability to sleep loss. Similarly, studies investigating other subregions of the hippocampus to repeated SD will be important for defining how distinct hippocampal subregions contribute to circuit-level adaptations to sleep loss.

In conclusion, this study reveals the subregional impact of sleep deprivation on hippocampal neuronal activation, which highlights the importance of spatially segregated analysis to understand the response to sleep loss. The reactivation of CA1 pyramidal neurons after repeated SD supports the premise that certain excitatory neuron populations are particularly susceptible to repeated sleep loss. Using the c-Fos-RiboTag system, we identified activity-driven gene expression alteration at a high resolution in SD-activated CA1 neurons and expanded the understanding of how SD could impair memory consolidation and progress toward chronic sleep restriction induced damages. Our research provides a detailed insight into hippocampal neurons responsive to SD and establishes a foundation for future molecular and functional investigations of neuron subsets activated by sleep loss. Identifying these subsets will help inform strategies to mitigate cognitive deficits associated with SD.

## Supplementary Information

Below is the link to the electronic supplementary material.

Supplementary Material 1.

Supplementary Material 2.

## Data Availability

The datasets generated and/or analyzed during the current study are available in the NCBI’s Gene Expression Omnibus repository, GEO Series accession GSE316227, [https://www.ncbi.nlm.nih.gov/geo/query/acc.cgi? acc=GSE316227] , and DEG accession GSE156925, [https://www.ncbi.nlm.nih.gov/geo/query/acc.cgi? acc=GSE156925] . Any additional information underlying this article will be shared on reasonable request to the corresponding author.

## Abbreviations

SD: Sleep deprivation
NSD: Non-sleep deprivation
CSR: Chronic sleep restriction
CA1: Cornus Ammonis 1
CA3: Cornus Ammonis 3
DG: Dentate Gyrus
IEG: Immediate early gene
ZT: Zeitgeber time
RT: Room temperature
h: Hour
min: Minute
AAV: Adeno-associated virus
4-OHT: 4-hydroxytamoxifen
i.p.: Intraperitoneally
IHC: Immunohistochemistry
PBS: Phosphate buffer saline
NP40: Nonidet P-40
TRAP: Target recombination in active populations
RiboTag: Ribosome tagging strategy
E: Expected overlap
RI: Reactivation index
TRAPseq: Translating ribosome affinity purification sequencing
ANOVA: Analysis of variance
DEG: Differentially expressed gene
FDR: False discovery rate
GO-BP: Gene ontology – biological process
KEGG: Kyoto encyclopedia of genes and genomes
SEM: Standard error of the mean

## References

[1] Zielinski MR, McKenna JT, McCarley RW. Functions and Mechanisms of Sleep. AIMS Neurosci. 2016;3(1):67–104. 28413828 10.3934/Neuroscience.2016.1.67 PMC5390528

[2] Adjaye-Gbewonyo D, Ng AE, Black LI. Sleep difficulties in adults: United States, 2020. 2022. 35792564

[3] Altevogt BM, Colten HR. Sleep disorders and sleep deprivation: an unmet public health problem. 2006. 20669438

[4] Killgore WD. Effects of sleep deprivation on cognition. Prog Brain Res. 2010;185:105–29. 21075236 10.1016/B978-0-444-53702-7.00007-5

[5] Zemla R, Basu J. Hippocampal function in rodents. Curr Opin Neurobiol. 2017;43:187–97. 28477511 10.1016/j.conb.2017.04.005 PMC5690575

[6] Grunwald T, Kurthen M. Novelty detection and encoding for declarative memory within the human hippocampus. Clin EEG Neurosci. 2006;37(4):309–14. 17073169 10.1177/155005940603700408

[7] Yassa MA, Stark CE. Pattern separation in the hippocampus. Trends Neurosci. 2011;34(10):515–25. 21788086 10.1016/j.tins.2011.06.006 PMC3183227

[8] Deuker L, Doeller CF, Fell J, Axmacher N. Human neuroimaging studies on the hippocampal CA3 region - integrating evidence for pattern separation and completion. Front Cell Neurosci. 2014;8:64. 24624058 10.3389/fncel.2014.00064 PMC3941178

[9] Kesner RP, Rolls ET. A computational theory of hippocampal function, and tests of the theory: new developments. Neurosci Biobehav Rev. 2015;48:92–147. 25446947 10.1016/j.neubiorev.2014.11.009

[10] Vecsey CG, Baillie GS, Jaganath D, Havekes R, Daniels A, Wimmer M, et al. Sleep deprivation impairs cAMP signalling in the hippocampus. Nature. 2009;461(7267):1122–5. 19847264 10.1038/nature08488 PMC2783639

[11] Prince TM, Wimmer M, Choi J, Havekes R, Aton S, Abel T. Sleep deprivation during a specific 3-hour time window post-training impairs hippocampal synaptic plasticity and memory. Neurobiol Learn Mem. 2014;109:122–30. 24380868 10.1016/j.nlm.2013.11.021 PMC3966473

[12] Walsh EN, Shetty MS, Diba K, Abel T. Chemogenetic Enhancement of cAMP Signaling Renders Hippocampal Synaptic Plasticity Resilient to the Impact of Acute Sleep Deprivation. eNeuro. 2023;10(1). 10.1523/ENEURO.0380-22.2022 PMC9829098 36635248

[13] Tudor JC, Davis EJ, Peixoto L, Wimmer ME, van Tilborg E, Park AJ, et al. Sleep deprivation impairs memory by attenuating mTORC1-dependent protein synthesis. Sci Signal. 2016;9(425):ra41. 27117251 10.1126/scisignal.aad4949 PMC4890572

[14] Havekes R, Park AJ, Tudor JC, Luczak VG, Hansen RT, Ferri SL et al. Sleep deprivation causes memory deficits by negatively impacting neuronal connectivity in hippocampal area CA1. Elife. 2016;5. 10.7554/eLife.13424 PMC4996653 27549340

[15] Raven F, Meerlo P, Van der Zee EA, Abel T, Havekes R. A brief period of sleep deprivation causes spine loss in the dentate gyrus of mice. Neurobiol Learn Mem. 2019;160:83–90. 29588221 10.1016/j.nlm.2018.03.018 PMC6420875

[16] Cirelli C, Tononi G. Effects of sleep and waking on the synaptic ultrastructure. Philos Trans R Soc Lond B Biol Sci. 2020;375(1799):20190235. 32248785 10.1098/rstb.2019.0235 PMC7209920

[17] de Almondes KM, Costa MV, Malloy-Diniz LF, Diniz BS. Insomnia and risk of dementia in older adults: Systematic review and meta-analysis. J Psychiatr Res. 2016;77:109–15. 27017287 10.1016/j.jpsychires.2016.02.021

[18] Simmonds E, Levine KS, Han J, Iwaki H, Koretsky MJ, Kuznetsov N, et al. Sleep disturbances as risk factors for neurodegeneration later in life. npj Dement. 2025;1(1):6.

[19] Konakanchi S, Raavi V, Ml HK, Shankar Ms V. Effect of chronic sleep deprivation and sleep recovery on hippocampal CA3 neurons, spatial memory and anxiety-like behavior in rats. Neurobiol Learn Mem. 2022;187:107559. 34808338 10.1016/j.nlm.2021.107559

[20] Giri S, Ranjan A, Kumar A, Amar M, Mallick BN. Rapid eye movement sleep deprivation impairs neuronal plasticity and reduces hippocampal neuronal arborization in male albino rats: Noradrenaline is involved in the process. J Neurosci Res. 2021;99(7):1815–34. 33819353 10.1002/jnr.24838

[21] Zhang F, Niu L, Zhong R, Li S, Le W. Chronic Sleep Disturbances Alters Sleep Structure and Tau Phosphorylation in AβPP/PS1 AD Mice and Their Wild-Type Littermates. J Alzheimers Dis. 2023;92(4):1341–55. 37038814 10.3233/JAD-221048

[22] Zhao B, Liu P, Wei M, Li Y, Liu J, Ma L, et al. Chronic Sleep Restriction Induces Aβ Accumulation by Disrupting the Balance of Aβ Production and Clearance in Rats. Neurochem Res. 2019;44(4):859–73. 30632087 10.1007/s11064-019-02719-2

[23] Jaworski J, Kalita K, Knapska E. c-Fos and neuronal plasticity: the aftermath of Kaczmarek’s theory. Acta Neurobiol Exp (Wars). 2018;78(4):287–96. 30624427

[24] Pompeiano M, Cirelli C, Tononi G. Immediate-early genes in spontaneous wakefulness and sleep: expression of c-fos and NGFI-A mRNA and protein. J Sleep Res. 1994;3(2):80–96. 10607112 10.1111/j.1365-2869.1994.tb00111.x

[25] Sherin JE, Shiromani PJ, McCarley RW, Saper CB. Activation of ventrolateral preoptic neurons during sleep. Science. 1996;271(5246):216–9. 8539624 10.1126/science.271.5246.216

[26] Cirelli C, Pompeiano M, Tononi G. Sleep deprivation and c-fos expression in the rat brain. J Sleep Res. 1995;4(2):92–106. 10607147 10.1111/j.1365-2869.1995.tb00157.x

[27] Terao A, Greco MA, Davis RW, Heller HC, Kilduff TS. Region-specific changes in immediate early gene expression in response to sleep deprivation and recovery sleep in the mouse brain. Neuroscience. 2003;120(4):1115–24. 12927216 10.1016/s0306-4522(03)00395-6

[28] Guenthner CJ, Miyamichi K, Yang HH, Heller HC, Luo L. Permanent genetic access to transiently active neurons via TRAP: targeted recombination in active populations. Neuron. 2013;78(5):773–84. 23764283 10.1016/j.neuron.2013.03.025 PMC3782391

[29] Sanz E, Bean JC, Carey DP, Quintana A, McKnight GS. RiboTag: Ribosomal Tagging Strategy to Analyze Cell-Type-Specific mRNA Expression In Vivo. Curr Protoc Neurosci. 2019;88(1):e77. 31216392 10.1002/cpns.77 PMC6615552

[30] Thompson CL, Wisor JP, Lee CK, Pathak SD, Gerashchenko D, Smith KA, et al. Molecular and anatomical signatures of sleep deprivation in the mouse brain. Front Neurosci. 2010;4:165. 21088695 10.3389/fnins.2010.00165 PMC2981377

[31] Lyons LC, Chatterjee S, Vanrobaeys Y, Gaine ME, Abel T. Translational changes induced by acute sleep deprivation uncovered by TRAP-Seq. Mol Brain. 2020;13(1):165. 33272296 10.1186/s13041-020-00702-5 PMC7713217

[32] Delorme J, Wang L, Kuhn FR, Kodoth V, Ma J, Martinez JD, et al. Sleep loss drives acetylcholine- and somatostatin interneuron-mediated gating of hippocampal activity to inhibit memory consolidation. Proc Natl Acad Sci U S A. 2021;118:32. 10.1073/pnas.2019318118 PMC8364159 34344824

[33] Vecsey CG, Peixoto L, Choi JH, Wimmer M, Jaganath D, Hernandez PJ, et al. Genomic analysis of sleep deprivation reveals translational regulation in the hippocampus. Physiol Genomics. 2012;44(20):981–91. 22930738 10.1152/physiolgenomics.00084.2012 PMC3472468

[34] Maciel R, Yamazaki R, Wang D, De Laet A, Cabrera S, Agnorelli C, et al. Is REM sleep a paradoxical state? Different neurons are activated in the cingulate cortices and the claustrum during wakefulness and paradoxical sleep hypersomnia. Biochem Pharmacol. 2021;191:114514. 33713640 10.1016/j.bcp.2021.114514

[35] Lein ES, Zhao X, Gage FH. Defining a molecular atlas of the hippocampus using DNA microarrays and high-throughput in situ hybridization. J Neurosci. 2004;24(15):3879–89. 15084669 10.1523/JNEUROSCI.4710-03.2004 PMC6729356

[36] Dobin A, Davis CA, Schlesinger F, Drenkow J, Zaleski C, Jha S, et al. STAR: ultrafast universal RNA-seq aligner. Bioinformatics. 2013;29(1):15–21. 23104886 10.1093/bioinformatics/bts635 PMC3530905

[37] Love MI, Huber W, Anders S. Moderated estimation of fold change and dispersion for RNA-seq data with DESeq2. Genome Biol. 2014;15(12):550. 25516281 10.1186/s13059-014-0550-8 PMC4302049

[38] Yu G, Wang LG, Han Y, He QY. clusterProfiler: an R package for comparing biological themes among gene clusters. Omics. 2012;16(5):284–7. 22455463 10.1089/omi.2011.0118 PMC3339379

[39] Shannon P, Markiel A, Ozier O, Baliga NS, Wang JT, Ramage D, et al. Cytoscape: a software environment for integrated models of biomolecular interaction networks. Genome Res. 2003;13(11):2498–504. 14597658 10.1101/gr.1239303 PMC403769

[40] Bindea G, Mlecnik B, Hackl H, Charoentong P, Tosolini M, Kirilovsky A, et al. ClueGO: a Cytoscape plug-in to decipher functionally grouped gene ontology and pathway annotation networks. Bioinformatics. 2009;25(8):1091–3. 19237447 10.1093/bioinformatics/btp101 PMC2666812

[41] Tang D, Chen M, Huang X, Zhang G, Zeng L, Zhang G, et al. SRplot: A free online platform for data visualization and graphing. PLoS ONE. 2023;18(11):e0294236. 37943830 10.1371/journal.pone.0294236 PMC10635526

[42] Delorme JE, Kodoth V, Aton SJ. Sleep loss disrupts Arc expression in dentate gyrus neurons. Neurobiol Learn Mem. 2019;160:73–82. 29635031 10.1016/j.nlm.2018.04.006 PMC6174112

[43] Kruijer W, Cooper JA, Hunter T, Verma IM. Platelet-derived growth factor induces rapid but transient expression of the c-fos gene and protein. Nature. 1984;312(5996):711–6. 6514007 10.1038/312711a0

[44] Lee H, GoodSmith D, Knierim JJ. Parallel processing streams in the hippocampus. Curr Opin Neurobiol. 2020;64:127–34. 32502734 10.1016/j.conb.2020.03.004 PMC8136469

[45] Neunuebel JP, Yoganarasimha D, Rao G, Knierim JJ. Conflicts between local and global spatial frameworks dissociate neural representations of the lateral and medial entorhinal cortex. J Neurosci. 2013;33(22):9246–58. 23719794 10.1523/JNEUROSCI.0946-13.2013 PMC3747988

[46] Gaine ME, Bahl E, Chatterjee S, Michaelson JJ, Abel T, Lyons LC. Altered hippocampal transcriptome dynamics following sleep deprivation. Mol Brain. 2021;14(1):125. 34384474 10.1186/s13041-021-00835-1 PMC8361790

[47] Colavito V, Fabene PF, Grassi-Zucconi G, Pifferi F, Lamberty Y, Bentivoglio M, et al. Experimental sleep deprivation as a tool to test memory deficits in rodents. Front Syst Neurosci. 2013;7:106. 24379759 10.3389/fnsys.2013.00106 PMC3861693

[48] Suzuki A, Sinton CM, Greene RW, Yanagisawa M. Behavioral and biochemical dissociation of arousal and homeostatic sleep need influenced by prior wakeful experience in mice. Proc Natl Acad Sci U S A. 2013;110(25):10288–93. 23716651 10.1073/pnas.1308295110 PMC3690840

[49] Vecsey CG, Wimmer ME, Havekes R, Park AJ, Perron IJ, Meerlo P, et al. Daily acclimation handling does not affect hippocampal long-term potentiation or cause chronic sleep deprivation in mice. Sleep. 2013;36(4):601–7. 23565007 10.5665/sleep.2556 PMC3612265

[50] Raven F, Heckman PRA, Havekes R, Meerlo P. Sleep deprivation-induced impairment of memory consolidation is not mediated by glucocorticoid stress hormones. J Sleep Res. 2020;29(5):e12972. 31845433 10.1111/jsr.12972 PMC7539978

[51] Sarma A, Ronde M, Smit S, Meerlo P, Havekes R. Does It Matter What Keeps You Awake? Effects of Two Different Sleep Deprivation Methods on Object-Location Memory and Hippocampal c-Fos Expression in Mice. J Sleep Res. 2025:e70079. 10.1111/jsr.70079 PMC12856129 40267993

[52] Wang L, Park L, Wu W, King D, Vega-Medina A, Raven F, et al. Sleep-dependent engram reactivation during hippocampal memory consolidation associated with subregion-specific biosynthetic changes. iScience. 2024;27(4):109408. 38523798 10.1016/j.isci.2024.109408 PMC10957462

[53] Lopez-Rodriguez F, Wilson CL, Maidment NT, Poland RE, Engel J. Total sleep deprivation increases extracellular serotonin in the rat hippocampus. Neuroscience. 2003;121(2):523–30. 14522011 10.1016/s0306-4522(03)00335-x

[54] Cirelli C, Pompeiano M, Tononi G. Neuronal gene expression in the waking state: a role for the locus coeruleus. Science. 1996;274(5290):1211–5. 8895474 10.1126/science.274.5290.1211

[55] Bacon TJ, Pickering AE, Mellor JR. Noradrenaline Release from Locus Coeruleus Terminals in the Hippocampus Enhances Excitation-Spike Coupling in CA1 Pyramidal Neurons Via β-Adrenoceptors. Cereb Cortex. 2020;30(12):6135–51. 32607551 10.1093/cercor/bhaa159 PMC7609922

[56] Liu Y, Cui L, Schwarz MK, Dong Y, Schlüter OM. Adrenergic Gate Release for Spike Timing-Dependent Synaptic Potentiation. Neuron. 2017;93(2):394–408. 28103480 10.1016/j.neuron.2016.12.039 PMC5267933

[57] Vanrobaeys Y, Peterson ZJ, Walsh EN, Chatterjee S, Lin LC, Lyons LC, et al. Spatial transcriptomics reveals unique gene expression changes in different brain regions after sleep deprivation. Nat Commun. 2023;14(1):7095. 37925446 10.1038/s41467-023-42751-z PMC10625558

[58] Yamazaki R, Wang D, De Laet A, Maciel R, Agnorelli C, Cabrera S et al. Granule cells in the infrapyramidal blade of the dentate gyrus are activated during paradoxical (REM) sleep hypersomnia but not during wakefulness: a study using TRAP mice. Sleep. 2021;44(12). 10.1093/sleep/zsab173 34245290

[59] Tayler KK, Tanaka KZ, Reijmers LG, Wiltgen BJ. Reactivation of neural ensembles during the retrieval of recent and remote memory. Curr Biol. 2013;23(2):99–106. 23246402 10.1016/j.cub.2012.11.019

[60] Li J, Jiang RY, Arendt KL, Hsu YT, Zhai SR, Chen L. Defective memory engram reactivation underlies impaired fear memory recall in Fragile X syndrome. Elife. 2020;9. 10.7554/eLife.61882 PMC7679137 33215988

[61] Gulmez Karaca K, Brito DVC, Kupke J, Zeuch B, Oliveira AMM. Engram reactivation during memory retrieval predicts long-term memory performance in aged mice. Neurobiol Aging. 2021;101:256–61. 33647524 10.1016/j.neurobiolaging.2021.01.019

[62] Pettit NL, Yap EL, Greenberg ME, Harvey CD. Fos ensembles encode and shape stable spatial maps in the hippocampus. Nature. 2022;609(7926):327–34. 36002569 10.1038/s41586-022-05113-1 PMC9452297

[63] Sadowski JH, Jones MW, Mellor JR. Sharp-wave ripples orchestrate the induction of synaptic plasticity during reactivation of place cell firing patterns in the hippocampus. Cell Rep. 2016;14(8):1916–29. 26904941 10.1016/j.celrep.2016.01.061 PMC4785795

[64] Girardeau G, Benchenane K, Wiener SI, Buzsáki G, Zugaro MB. Selective suppression of hippocampal ripples impairs spatial memory. Nat Neurosci. 2009;12(10):1222–3. 19749750 10.1038/nn.2384

[65] Giri B, Kinsky N, Kaya U, Maboudi K, Abel T, Diba K. Sleep loss diminishes hippocampal reactivation and replay. Nature. 2024;630(8018):935–42. 38867049 10.1038/s41586-024-07538-2 PMC11472378

[66] Daskin E, Van S, Hafner AS. Local Protein Synthesis at Synapses: A Driver for Synapse Diversification. J Neurochem. 2025;169(11):e70308. 41299820 10.1111/jnc.70308 PMC12657692

[67] Zaki Y, Cai DJ. Memory engram stability and flexibility. Neuropsychopharmacology. 2024;50(1):285–93. 39300271 10.1038/s41386-024-01979-z PMC11525749

[68] He Y, Sun M, Qu M, Lu Y, Yang H, Wang R, et al. Brain Insulin Signaling Pathway Regulation of Hippocampal Neuroplasticity in Neurocognitive Disorders: Mechanisms and Therapeutic Implications. J Integr Neurosci. 2025;24(8):39446. 40919624 10.31083/JIN39446

[69] Grillo CA, Piroli GG, Lawrence RC, Wrighten SA, Green AJ, Wilson SP, et al. Hippocampal Insulin Resistance Impairs Spatial Learning and Synaptic Plasticity. Diabetes. 2015;64(11):3927–36. 26216852 10.2337/db15-0596 PMC4613975

[70] Giese KP. GSK-3: a key player in neurodegeneration and memory. IUBMB Life. 2009;61(5):516–21. 19391164 10.1002/iub.187

[71] Lauretti E, Dincer O, Praticò D. Glycogen synthase kinase-3 signaling in Alzheimer’s disease. Biochim Biophys Acta Mol Cell Res. 2020;1867(5):118664. 32006534 10.1016/j.bbamcr.2020.118664 PMC7047718

[72] Nozal V, Martinez A. Tau Tubulin Kinase 1 (TTBK1), a new player in the fight against neurodegenerative diseases. Eur J Med Chem. 2019;161:39–47. 30342424 10.1016/j.ejmech.2018.10.030

[73] Wang X, Chen H, Tang T, Zhan X, Qin S, Hang T et al. Chronic Sleep Deprivation Altered the Expression of Memory-Related Genes and Caused Cognitive Memory Dysfunction in Mice. Int J Mol Sci. 2024;25(21). 10.3390/ijms252111634 PMC11546330 39519186

