# Supplementary material for: Repeated sleep deprivation selectively reactivates hippocampal CA1 pyramidal neurons
Source: Mol Brain. 2026 Apr 1;19:39. doi: 10.1186/s13041-026-01298-y (PMC13169576; doi:10.1186/s13041-026-01298-y)
Supplement: Supplementary file 1 — Supplementary Material 1. [file 13041_2026_1298_MOESM1_ESM.pdf]

# Figure S1

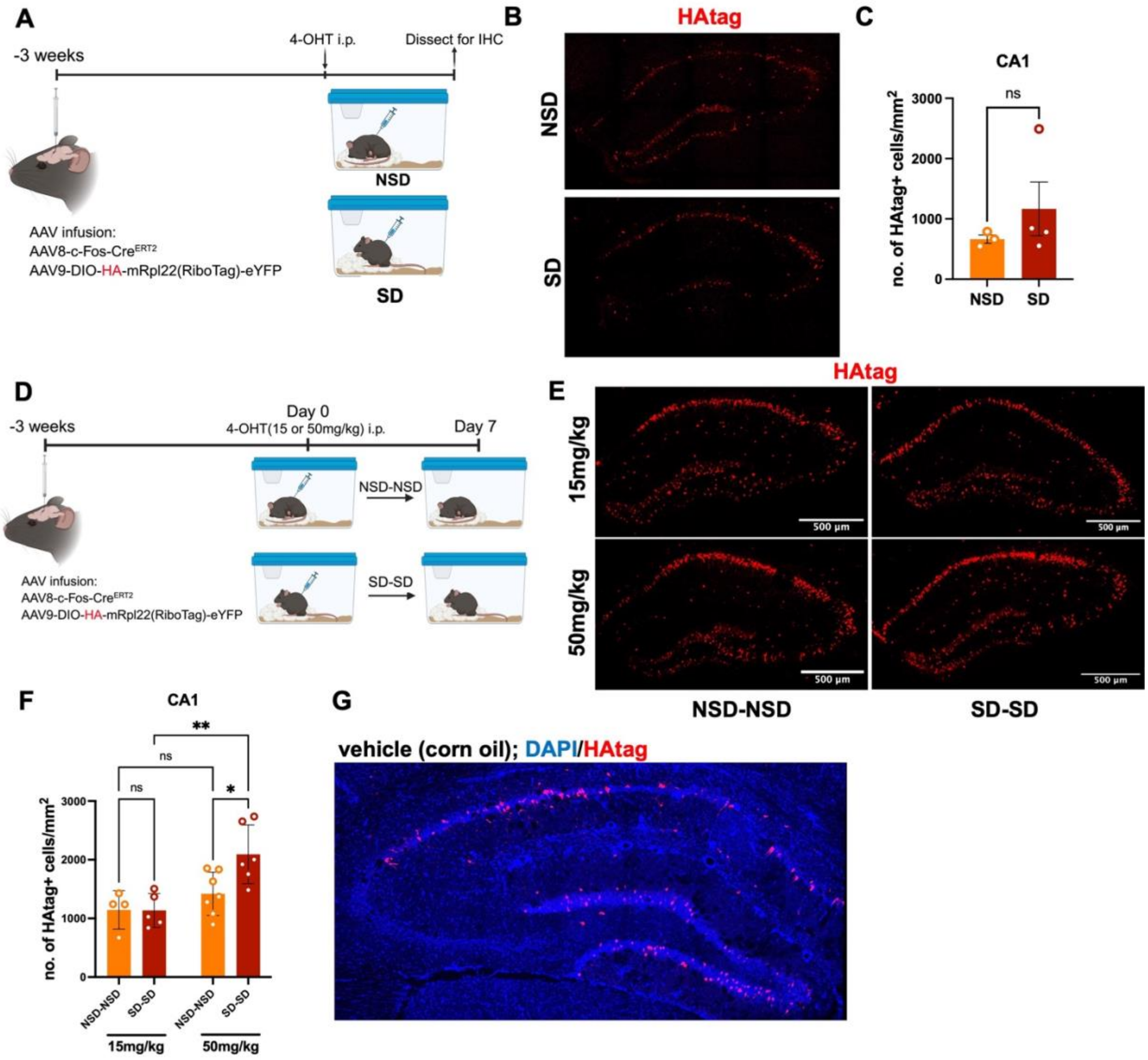


**Figure S1**. **Supplement data for RiboTag expression following single and repeated SD. A**) Schematic of the c-Fos promoted RiboTag expression following 5h of SD. **B**) Representative image of RiboTag (HAtag) expression in NSD/SD hippocampus. **C**) Comparison of area normalized HAtag+ expression between NSD (n=3) and SD (n=4) groups within area CA1. Unpaired t-test, NSD=48.28±6.60, SD=77.92±19.24, t(6)=0.7073, $p>0.05$. **D**) Timeline of the CA1 neuron labeling using the cFos-RiboTag strategy (whole hippocampus infusion) with repeated sleep deprivation. **E**) Representative images of neuron labeling shown as RiboTag

(HAtag) expression in the NSD and SD hippocampus injected with 15mg/kg or 50mg/kg 4-OHT. **F**) Comparison of neuronal labeling efficacy in hippocampal CA1: area normalized HAtag+ cells in NSD and SD groups injected with 15mg/kg and 50mg/kg of 4-OHT, n= 4-7 mice in each group. Two-way ANOVA test performed. Data shown as mean ± SEM. D: No significant effect of repeated sleep loss ($F(1,18)=3.815$, $p=0.0665$) but a significant effect of 4-OHT dose ($F(1,18)=13.21$, $p=0.0019$) and a significant interaction of repeat manipulation and 4-OHT dose ($F(1,18)=4.049$, $p=0.0594$). Bonferroni post hoc: 15mg/kg: NSD=1145.54±168.88, SD=1209.69±135.90, $t(18)=0.0384$, $p>0.9999$; 50mg/kg: NSD=1420.40±139.43, SD=2092.38±328.09, $t(18)=3.106$, $p=0.0366$. 15mg/kg vs 50mg/kg: p(NSD)>0.9999, p(SD)=0.0044. **G**) Representative image of background RiboTag expression (~200/mm2) in negative control mice injected with vehicle (corn oil) rather than 4-OHT.

## Figure S2

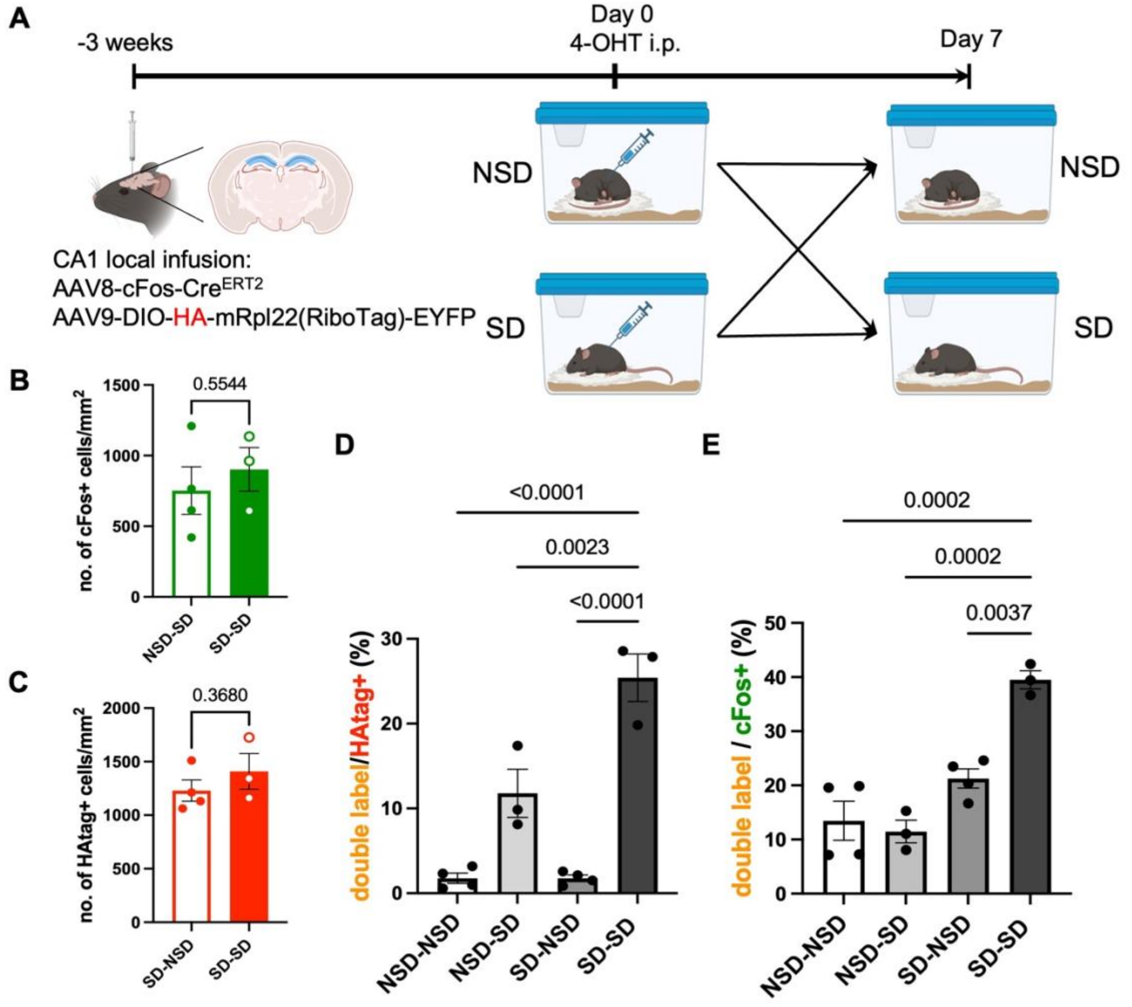


**Figure S2. Repeated SD leads to robust CA1 pyramidal neuron reactivation. A**) Diagram of neuronal reactivation comparison among NSD-NSD, NSD-SD, SD-NSD, SD-SD conditions. **B**) Area normalized c-Fos expression in hippocampal CA1 between NSD-SD (n=4) and SD-SD (n=3) groups. NSD-SD=752.0±168.2; SD-SD= 902.3±154.5. **C**) Neuronal labeling (HAtag expression) in hippocampal CA1 between SD-NSD (n=4) and SD-SD (n=4) groups. SD-NSD= 1229.7±98.74; SD-SD= 1409.6±155.5. Unpaired two-tailed t-test performed for B, C). **D, E**) Neuronal reactivation: percentage (%) of double labeled cells / HAtag+ cells (D) and double labeled cells / c-Fos+ cells (E) overlap in NSD-NSD (n=4), NSD-SD (n=3), SD-NSD (n=4), and SD-SD (n=3) CA1. D: NSD-NSD=1.76±0.60, NSD-SD=11.78±2.85, SD-NSD=1.77±0.37, SD-

SD=25.42±2.81. E: NSD-NSD=13.45±3.61, NSD-SD=11.47±2.09, SD-NSD=21.26±1.78, SD-SD=39.51±1.67. One-way ANOVA performed for D, E). Data shown as mean ± SEM, p values indicated in figures.

# Figure S3

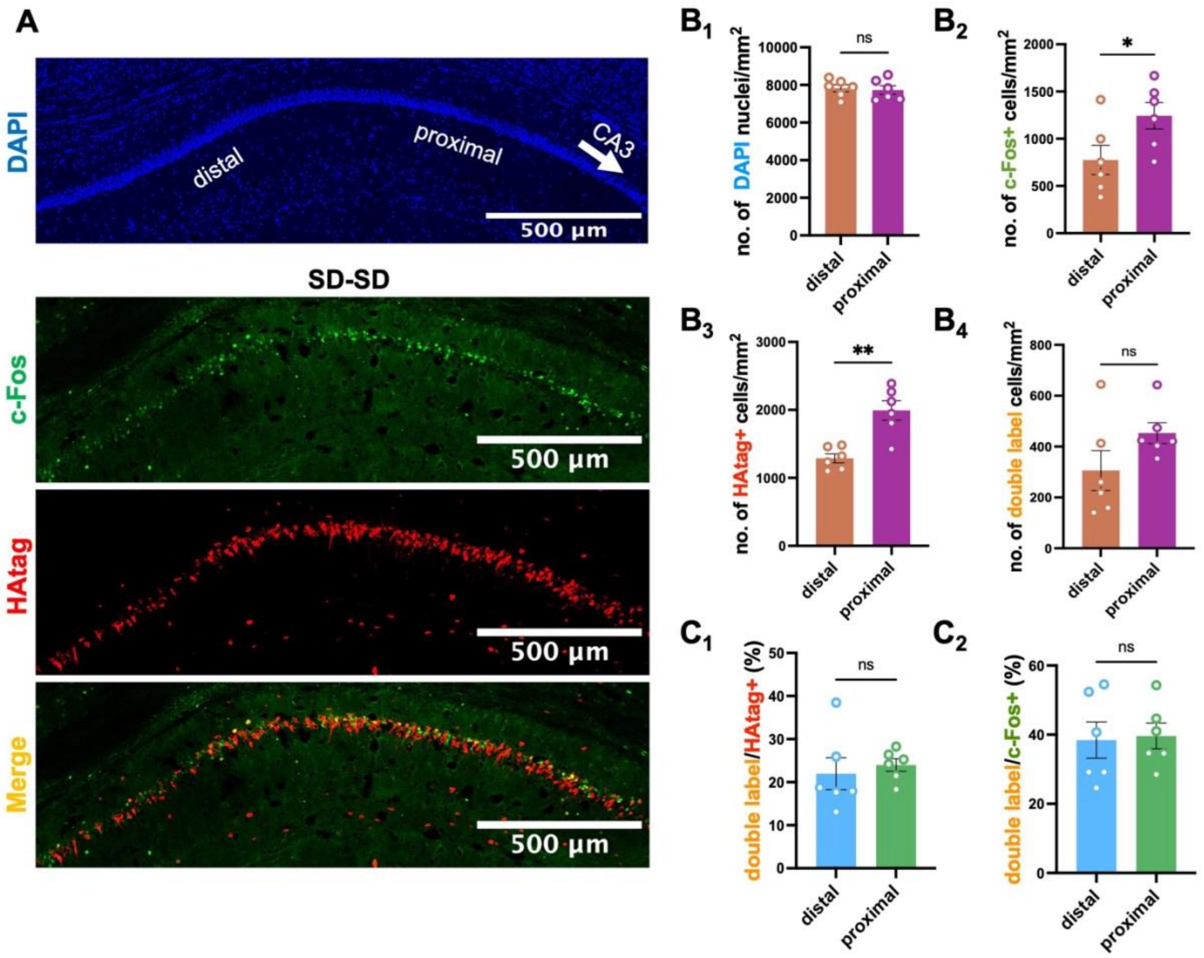


**Figure S3**. **Proximal CA1 shows greater c-Fos expression than distal CA1 after SD. A**) Representative image of DAPI, cFos+ and HAtag+ cells in distal vs proximal CA1 in SD-SD group. **B**) Comparison of area normalized DAPI nuclei (1), c-Fos+ (2), HAtag+ (3), and double labeled (4) cells between distal vs proximal CA1 in SD-SD mice (n=6). Unpaired t-tests. Data shown as mean ± SEM. B1) dCA1=7827.39±190.20, pCA1=7732.98±227.45, t(10)=0.32, *p*=0.7576. B2) dCA1= 776.04±154.53, pCA1=1243.15±140.26, t(10)=2.24, *p*=0.0491. B3) dCA1=1287.53±65.68, pCA1=1993.05±142.29, t(10)=4.50, *p*=0.0011. B4) dCA1=305.80±78.63, pCA1=452.91±41.19, t(10)=1.66, *p*=0.1285. **C**) Comparison of neuronal reactivation rate: percentage (%) of double labeled cells / HAtag+ cells (1) and double labeled cells / c-Fos+ cells

(2) between distal vs proximal CA1 in SD-SD mice. Unpaired t-tests, data shown as mean ± SEM. C1 (%): dCA1=21.97±3.71, pCA1=23.99±1.46, $t(10)=0.51$, $p=0.6230$. C2 (%): dCA1=38.43±5.24, pCA1=39.62±3.73, $t(10)=0.18$, $p=0.8572$.

## Figure S4

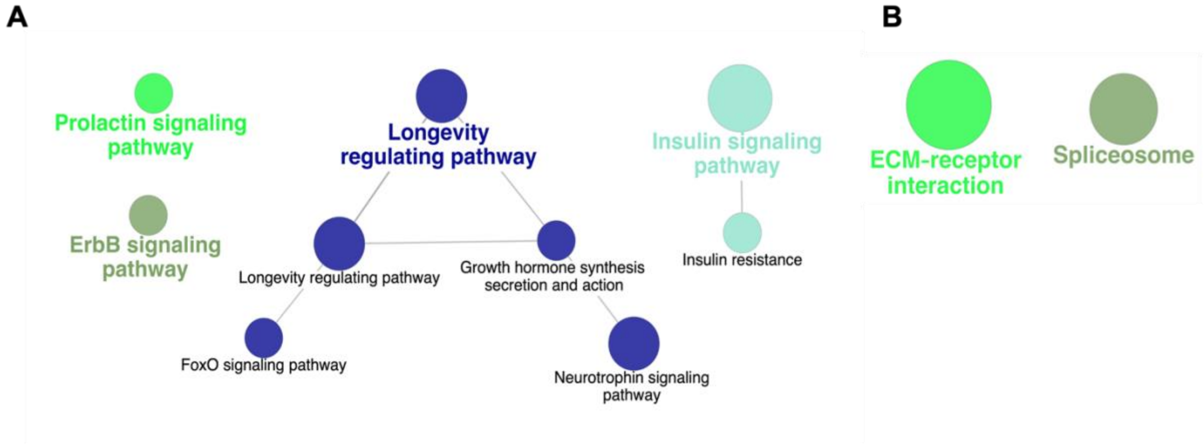


**Figure S4. fosTRAP-seq biological function enrichment analysis. A, B**) Map of KEGG pathways associated with upregulated DEGs (A) and downregulated DEGs (B) after sleep deprivation in SD-sensitive hippocampal CA1 neurons (adjusted p<0.05). Full details of KEGG pathways see additional file2: Table S5.

# Figure S5

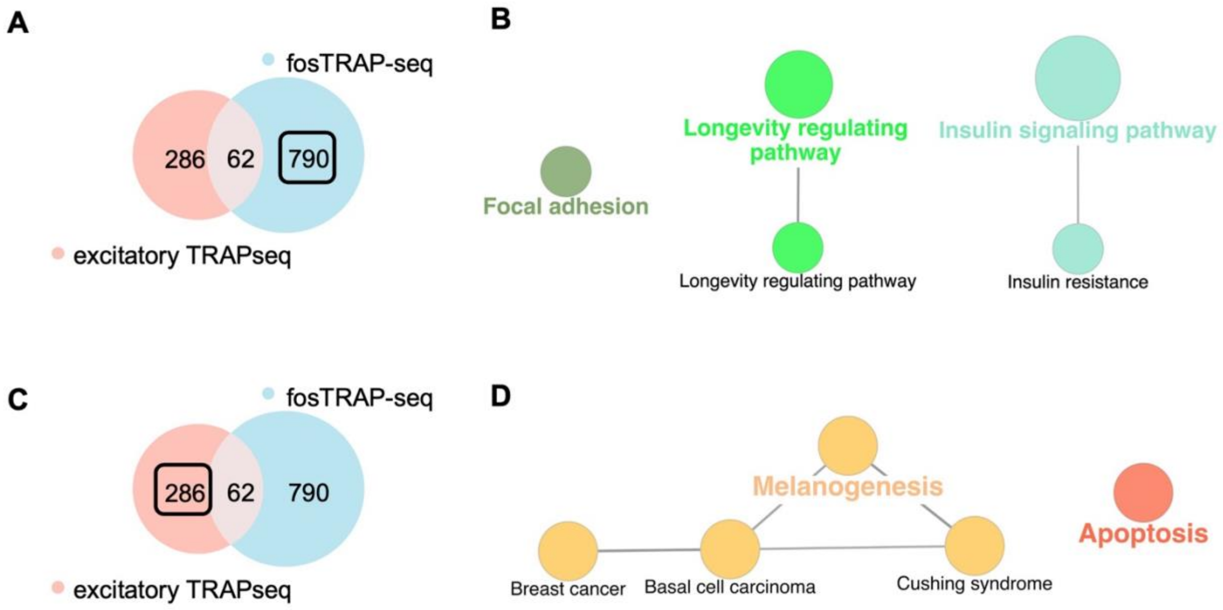


**Figure S5. Comparison between fosTRAP-seq and excitatory TRAPseq. A**) Venn diagram representing DEGs specific to fosTRAP-seq analysis. **B**) KEGG pathways (adjusted p<0.05) associated with fosTRAP-seq unique DEGs. **C**) Venn diagram representing DEGs specific to excitatory TRAP-seq analysis. **D**) KEGG pathways (adjusted p<0.05) associated with excitatory TRAPseq unique DEGs. Full details see additional file2: Table S8.
